# TRP (transient receptor potential) ion channel family: structures, biological functions and therapeutic interventions for diseases

**DOI:** 10.1038/s41392-023-01464-x

**Published:** 2023-07-05

**Authors:** Miao Zhang, Yueming Ma, Xianglu Ye, Ning Zhang, Lei Pan, Bing Wang

**Affiliations:** 1 School of Pharmacy, Shanghai University of Traditional Chinese Medicine, Shanghai, 201203 China; 2 Experiment Center for Science and Technology, Shanghai University of Traditional Chinese Medicine, Shanghai, 201203 China; 3 The Center for Microbes, Development and Health; Key Laboratory of Molecular Virology and Immunology, Institut Pasteur of Shanghai, Chinese Academy of Sciences, Shanghai, 200031 China; 4 Institute of Interdisciplinary Integrative Medicine Research, Shanghai University of Traditional Chinese Medicine, Shanghai, 201203 China; 5 CAS Center for Excellence in Biotic Interactions, University of Chinese Academy of Sciences, Beijing, 100049 China; 6 Center for Pharmaceutics Research, Shanghai Institute of Materia Medica Chinese Academy of Sciences, Shanghai, 201203 China

**Keywords:** Biochemistry, Diseases

## Abstract

Transient receptor potential (TRP) channels are sensors for a variety of cellular and environmental signals. Mammals express a total of 28 different TRP channel proteins, which can be divided into seven subfamilies based on amino acid sequence homology: TRPA (Ankyrin), TRPC (Canonical), TRPM (Melastatin), TRPML (Mucolipin), TRPN (NO-mechano-potential, NOMP), TRPP (Polycystin), TRPV (Vanilloid). They are a class of ion channels found in numerous tissues and cell types and are permeable to a wide range of cations such as Ca^2+^, Mg^2+^, Na^+^, K^+^, and others. TRP channels are responsible for various sensory responses including heat, cold, pain, stress, vision and taste and can be activated by a number of stimuli. Their predominantly location on the cell surface, their interaction with numerous physiological signaling pathways, and the unique crystal structure of TRP channels make TRPs attractive drug targets and implicate them in the treatment of a wide range of diseases. Here, we review the history of TRP channel discovery, summarize the structures and functions of the TRP ion channel family, and highlight the current understanding of the role of TRP channels in the pathogenesis of human disease. Most importantly, we describe TRP channel-related drug discovery, therapeutic interventions for diseases and the limitations of targeting TRP channels in potential clinical applications.

## Introduction

Transient receptor potential (TRP) channels, which were first discovered in 1969,^1^ they are multimodal ion channels that act as sensors of chemically toxic and physical stimuli.^2^ These channels are widely distributed in various tissues and play a variety of roles.^3^ Despite having been studied for a long time, the significance of TRP channels remains unclear. In addition, changes in TRP channels have been observed in a variety of diseases.^4^ More importantly, the regulation of TRP channels can be exploited to prevent or treat diseases. In this review, we present a historical perspective on TRP channels. We then discuss the structure and function of TRP channels and summarize their different types, focusing on their key roles in various physiological and pathological conditions, particularly in pain, inflammatory bowel disease (IBD) and its complications, respiratory diseases, neurological disorders, and cardiovascular diseases (CVDs). We also highlight therapeutic interventions, including physical and pharmacological ones, regarding TRP channels. Finally, we conclude this review with a summary of frequent adverse events that occur during treatments.

## History of TRP channels

**Fig. 1:**
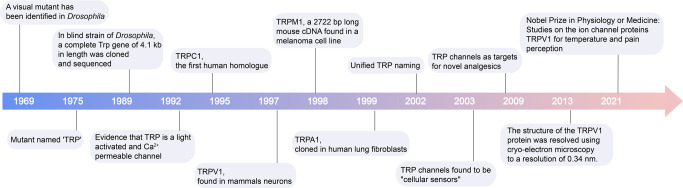
The milestones of the discoveries and research advances of TRPs. After the first discovery of TRPs in 1969, multiple classes of TRPs were identified in different species. With the development of sequencing technology, the biological properties and functions of TRPs have been extensively studied

TRP channels were first proposed over 50 years ago (Fig. 1). In 1969, Cosens et al. identified a visual mutant in *Drosophila*.^1^ The mutant showed a transient rather than a continuous response to bright light stimuli. Minke et al. were the first to name the mutant “transient receptor potential” (*trp*) in 1975 based on its electrophysiological phenotype.^5^ In 1989, Montell et al.^6^ and Wong et al.^7^ successively cloned the *trp* gene and recognized it as a transmembrane protein. Since then, researchers have focused on expanding the understanding of *trp*.

Minke’s group was the first to establish a link between *trp* mutants and Ca^2+^.^8^ Shortly thereafter, Minke and Selinger confirmed that the trp protein is a plasma membrane component (or part of one) that oscillates between Ca^2+^-transporting and non-transporting states.^9^ A strong compelling evidence demonstrated that TRP is a light-activated and Ca^2+^, permeable channel, as discovery by Hardie and Minke.^10^ Meanwhile, Kelly and colleagues observed that TRP-like proteins are typical of voltage-gated channels.^11^ They also identified the ankyrin repeats in the known amino acid (aa) sequence of TRP. After the 1990s, the presence of TRPs was confirmed in various types of cell lines.^12,13^ These reports provided a glimpse into the biological functions of TRPs. In addition, TRPs are widely present in healthy human tissues.

In 2002, Montell et al.^14^ and Clapham et al.^15^ undertook work to unify the TRP nomenclature. The 28 channel subunit genes were subdivided into seven subfamilies, namely, TRPA (ankyrin), TRPC (canonical), TRPM (melastatin), TRPML (mucolipin), TRPN (NO-mechano-potential, NOMP), TRPP (polycystin) and TRPV (vanilloid), based on their gene sequences. Mammals are represented in all subfamilies.^16^ Research on diseases based on TRPs has received a great deal of attention in the last two decades. In particular, the development of molecular biology technologies has improved the understanding of TRPs.

## TRP ion channel family

### Classifications of TRP

**Fig. 2:**
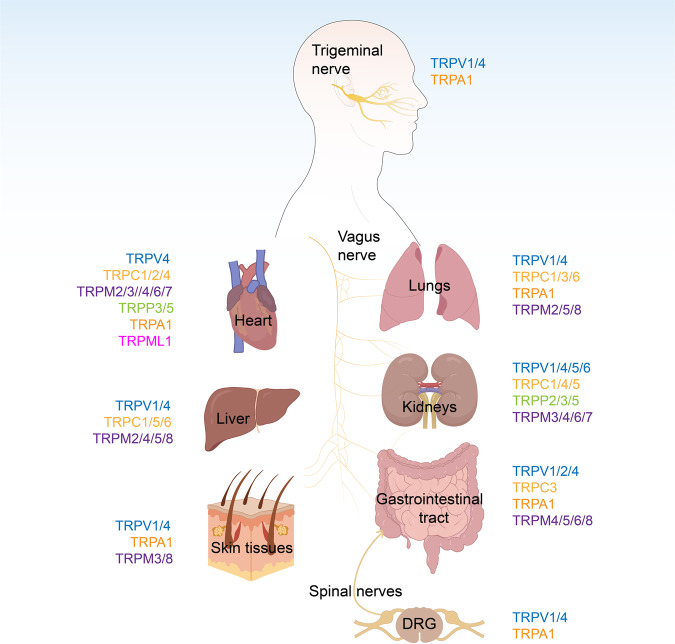
Distribution of TRP channels in the human body. TRP channels are widely distributed in human organs, such as lung, liver, skin, nerves, and intestine. The different colors of the words represent the different TRP families. Created with BioRender.com

Based on sequence and topological differences,^6^ mammalian TRPs can be categorized into the following seven subfamilies: group 1 with five subfamily members (TRPC, TRPV, TRPM, TRPN, and TRPA), and group 2 with two subfamilies (TRPP and TRPML) (Table 1 and Fig. 2).^17^ An additional subfamily, TRPY, consists of yeast TRPs, which are distantly related to group 1 and group 2 TRPs.^18^ The first and second groups both have six transmembrane structural domains and are permeable to cations. *TRPA*, *TRPV*, and *TRPC* channels contain ankyrin repeat sequences in their intracellular N-terminal structural domains, whereas *TRPC* and *TRPM* subfamilies possess a proline-rich “TRP structural domain” in the C-terminal region near the presumed transmembrane segment.^19^ Group 2 channels also have high sequence homology in their transmembrane structural domains and are only distally related to the genes of group 1 channels because they contain a large lumenal/extracellular domain between transmembrane helix 1 (S1) and S2.^20^

**Table 1:** The unique characteristics and functions of different types of TRP channels

Classification		Chromosomal localization	Distribution	Function	Ref.
TRPC	TRPC1	3q22–q24	Heart, brain, testis, ovary, liver, spleen, kidney, skeletal muscle, lung	Myoblast fusion; Inhibition of tumor growth and metastasis; Immune regulation; Vasoconstriction	^617,618^
	TRPC2	7, 50.0 cM	Brain, testis, sperm, heart, brain, vomeronasal sensory organ	Olfaction; Regulation of thyroid function	^619,620^
	TRPC3	4q27	Brain (mainly), heart, muscle, ovary, colon, small intestine, lung, prostate, placenta, testis	Multifunctional cell sensors; Immune regulation; Cell depolarization; Vasodilation	^621,622^
	TRPC4	13q13.1–q13.2	Brain, endothelia, adrenal gland, retina, testis, placenta, kidney, vascular system	Angiogenesis; Promotion of migration and proliferation of cancer cells	^623,624^
	TRPC5	Xq23	Brain (mainly), liver, kidney, testis, uterus, vascular system	Angiogenesis, Regulation of blood pressure; Endothelium-dependent contraction; Promotion of extracellular vesicle formation	^625–627^
	TRPC6	11q21–q22	Lung, brain, muscle, placenta, ovary	Protection of neurons; Immune regulation; Development of glomerular injury	^628,629^
	TRPC7	5q31.2	Heart, lung, eye, brain, spleen, testis	Cardiac hypertrophy?	^630^
TRPV	TRPV1	17p13.3	TG, DRG, neurons, urinary bladder, testis	Neurodepolarization; Thermosensitivity; Mechanosensitivity	^631,632^
	TRPV2	17p11.2	DRG, spinal cord, brain, spleen, intestine	Thermosensitivity; Mechanosensitivity	^239,633^
	TRPV3	17p13.3	TG, DRG, spinal cord, brain, keratinocytes, tongue	Thermosensitivity	^258,631^
	TRPV4	12q24.1	DRG, kidney, lung, spleen, testis, keratinocytes, heart, liver, endothelia	Maintenance of cellular osmotic homeostasis; Thermosensitivity; Thermoregulation; Mechanosensitivity	^633,634^
	TRPV5	7q35	Kidney, intestine, pancreas, placenta	Regulation of calcium channels	^635^
	TRPV6	7q33–q34	Small intestine, pancreas, placenta, kidney	Calcium regulation; Maintenance of skeletal integrity	^636,637^
TRPA1		8q13	DRG, TG, hair cells, ovary, spleen, testis, lung, heart, brain, pancreas	Thermosensitivity; Nociception	^93,215^
TRPM	TRPM1	15q13–q14	Brain, melanosomes, eye, skin	Depolarization of the bipolar cells; Suppression of melanoma metastasis	^638^
	TRPM2	21q22.3	Brain, bone marrow, spleen, heart, lung	Core body temperature sensation; Oxidative sensation; Insulin secretion; Immune response; Maintenance of mitochondrial function	^103,639^
	TRPM3	9q21.11	Kidney, brain, pituitary, pancreas, eye, heart, DRG	Glucose homeostasis; Heat sensation and inflammatory pain; Regulation of insulin release	^640,641^
	TRPM4	19q13.33	Prostate, colon, heart, kidney, testis, skin, pancreas, placenta, liver, intestines, thymus, spleen	Regulation of calcium oscillations after T cell activation; Prevention of cardiac conduction disorders; Regulation of smooth muscle contraction; Immune response	^163,642^
	TRPM5	11p15.5	Intestine, liver, lung, taste cells	Modulation of insulin secretion and sensory transduction in taste cells; Induced depolarization of cell membranes; Immune response; Constriction of cerebral arteries	^643^
	TRPM6	9q21.13	Kidney, small intestines, heart	Magnesium uptake and homeostasis in kidney and intestine	^644^
	TRPM7	15q21	Kidney, heart, pituitary, bone, adipose	Magnesium and calcium homeostasis; Cell viability	^645^
	TRPM8	2q37.2	DRG, TG, prostate, liver, lung, skin, pancreas, colon	Cold sensation	^116,646^
TRPN		/	Ear, eye	Hearing	^25^
TRPP	TRPP2	4q21–q23	Widely expressed, kidney	Ca^2+^-permeable	^647^
	TRPP3	10q24	Kidney, heart, testis, cardiac, neurons	Ca^2+^-permeable; Taste reception	^124,648–650^
	TRPP5	5q31	Testis, heart, kidney	Ca^2+^ signaling	^649,650^
TRPML	TRPML1	19p13.2–p13.3	Brain, heart, skeletal muscle	Organelle biogenesis; Regulation of lysosomal hydrolysis; Regulated sorting of lipids	^651^
	TRPML2	1p22	/	B-lymphocyte development; Immune response	^652^
	TRPML3	1p22.3	Cochlear hair cells	Cell depolarization; Overload with Ca^2+^	^653,654^

*DRG* dorsal root ganglia, *TG* trigeminal ganglia

#### TRPC

TRPC channels were the first identified members of the TRP family.^21^ TRPC channels are nonselective cation channels.^22^ They range from a few tens to a few hundred kilodaltons.^23^ In 1995, the full sequence of the first human homolog (TRPC1) was reported.^24^ Since then, seven mammalian TRPC proteins (TRPC1–7) have been described.^14^ Based on aa similarities, mammalian TRPCs fall into four subsets: TRPC1, TRPC2, TRPC3/6/7, and TRPC4/5.^25^ TRPC channel proteins are expressed in excitable and non-excitable cells.^26^ TRPCs have a broad tissue-specific distribution and are therefore involved in various pathophysiological functions.^27^

#### TRPV

TRPV channels are a part of the TRP channel superfamily and named for their sensitivity to vanilloid and capsaicin.^28,29^ TRPVs are divided into two types.^30^ TRPV1–4 can be thermally activated and are therefore called thermal TRP channels.^31^ One of the first clues toward understanding the functional diversity of TRPV channels came from the structural studies of the N-terminal ankyrin repeat domain.^32^ In most tissues, these domains serve as sensors for different pain stimuli (heat, pressure, and pH) and contribute to the homeostasis of electrolytes, the maintenance of barrier functions, and the development of macrophages.^33,34^ Given fundamental role in a multitude of physiological and pathophysiological processes, TRPV channels are promising targets for drug development.

#### TRPA

In 1999, the first human TRPA protein, TRPA1, was discovered during a screening of downregulated genes following the oncogenic transformation of fibroblasts.^35^ TRPA1 is the only TRPA protein present in humans.^36,37^ TRPA1 was previously called ANKTM1 because the protein consists of numerous N-terminal ankyrin repeats.^38^ TRPA1 is a sensor for diverse noxious external stimuli such as intense cold, irritating compounds, mechanical stimuli, reactive chemicals, and endogenous signals associated with cellular damage.^39^ This diversity of functions and expression in nociceptive nerve fibers, epithelial cells, and various other cells make this channel relevant to a wide range of diseases and an attractive therapeutic target.^40^

#### TRPM

Since the first cloning of TRPM1 in 1998, tremendous progress has been made in the identification of novel members of the TRPM subfamily and their functions.^41^ The TRPM subfamily consists of eight members; TRPM1–TRPM8.^42^ TRPMs have been involved in several physiological and pathological processes, including cellular proliferation,^43^ temperature sensing,^44^ vascular development,^45^ cancer progression,^46^ neurological diseases,^47^ endothelial dysfunction,^48^ inflammation,^49^ type II diabetes,^50^ and many other processes. TRPM channels possess a large cytosolic domain consisting of 732 and 1611 aa for each subunit, making them the largest members of the TRP superfamily.^51^

#### TRPN

The TRPN subfamily was named based on the founding member, *Drosophila* NOMPC.^52^ Mammals do not encode any TRPN members.^53^ TRPNs are found in worms, flies, and zebrafish.^54^ Genetic screening, calcium imaging, and electrophysiological analysis have identified TRPN as a mechanically gated channel in eukaryotes.^55^ A TRPN channel protein is essential for sensory transduction in insect mechanosensory neurons and in vertebrate hair cells.^56^ However, without a molecular marker for the protein, its exact location and role in transduction are uncertain.

#### TRPP and TRPML

TRPP and TRPML belong to group 2 of TRP channels and have limited homology similarity to group 1. Furthermore, the large loop between transmembrane domains (TMD) 1 and 2, which discriminates them from group 1.^25^ Invertebrate TRPPs have been described in *C. elegans*,^57^ *Drosophila* (AMO),^58^ and the sea urchin (suPC2).^59^ The three human genes encoding for the TRPP protein family are polycystic kidney disease 2 (*PKD2* and *TRPP2*), PKD2-like 1 (*PKD2L1* and *TRPP3*), and PKD2-like 2 (*PKD2L2* and *TRPP5*).^17^ TRPPs may comprise the most primitive TRP subfamily given that as microbial homologs of TRPP2 are found in yeast.^60^ In addition to functioning as a cation influx channel, TRPP2 is also localized in the endoplasmic reticulum (ER) membrane where it is proposed to serve as a new type of Ca^2+^-release channel.^61^

The mucolipin family of the ion channel TRP superfamily (TRPML) includes three members: TRPML1, TRPML2, and TRPML3.^62^ The molecular weights of TRPMLs are around 65 kDa.^63^ TRPML1, was cloned during the search for the genetic determinants of the lysosomal storage disease MCOLN.^64^ Mutations of this protein are characterized by severe neurodegeneration. Defects in TRPML function are predicted to have important effects on organelle acidification, vesicle fusion, endosome maturation, and signaling; thus, suggesting that this protein family plays a key role in normal and pathological conditions.^65,66^

### Structures of TRP channels

**Fig. 3:**
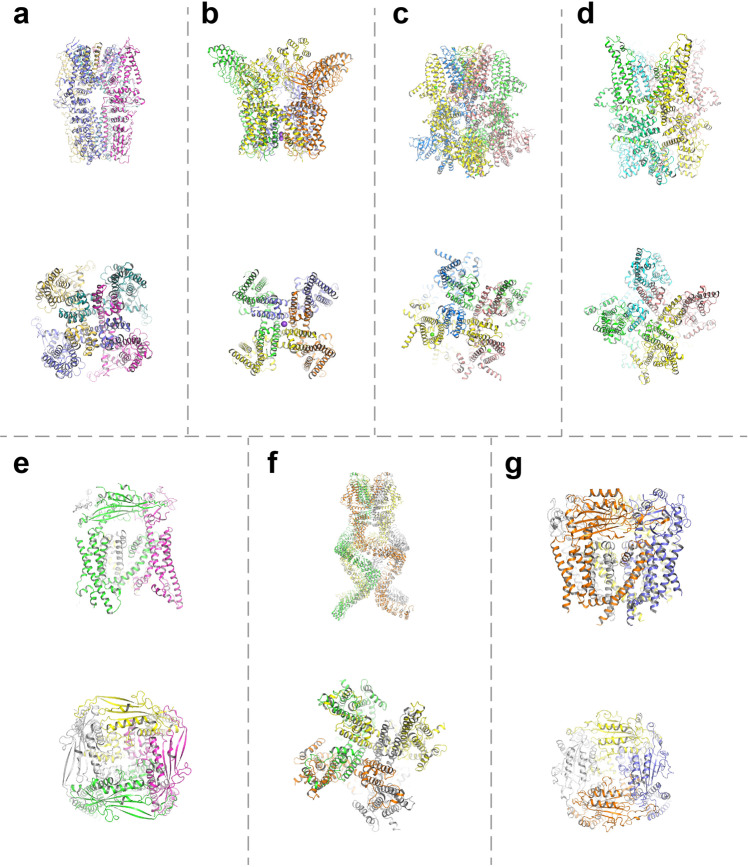
Structure of TRP channels. Representative structures for each TRP subfamily. In each small figure, the top one is the side view and the bottom one is the top view. **a** TRPA1 (PDB ID: 6PQP); **b** TRPV1 (PDB ID: 7LP9); **c** TRPM8 (PDB ID: 7WRB); **d** TRPC3 (PDB ID: 6CUD); **e** TRPML1 (PDB ID: 7SQ8); **f** TRPN (PDB ID: 5VKQ); **g** TRPP2 (PDB ID: 5T4D)

Since the discovery of TRPs, researchers have systematically analyzed their structure TRPs through technical means (Table 2 and Fig. 3). The progressive development of sequencing and analytical techniques has revealed several key structural features of TRPs. TRP channels have six transmembrane spanning domains (S1–S6), with a pore-forming loop between S5 and S6, and both the C and N termini are located intracellularly.^67^ The cytoplasmic end of the S6 helix forms to form the lower gate, which opens and closes to regulate cation entry into the channel. The S1–S4 domains may gate the pore in response to ligand binding, but the paucity of positively charged arginine in the S4 helix indicates the weak voltage sensitivity of TRP channels. All elements outside the S5–S6 region provide means of either subunit association. They also act as a linker for the elements that control the gating. Cryo-electron microscopy (cryo-EM) confirmed that TRPs have a structure similar to that of voltage-gated ion channels.^68^ Most TRPs form functional channels as homotetramers, but heteromultimerization is frequently observed.^69^ This condition creates a potential problem for drug discovery efforts because heteromultimers (which are difficult to recreate in heterologous expression systems) may have distinct pharmacological properties.

**Table 2:** Structures of the TRP channels

Classification		Receptor type	Species	Ligand	Released year	Resolution (Å)	PDB ID	Ref.
TRPC	TRPC3	Short transient receptor potential channel 3	Human	(2S)-3-{[(S)-(2-aminoethoxy)(hydroxy)phosphoryl]oxy}-2-(hexanoyloxy)propyl hexanoate; (2R)-3-hydroxypropane-1,2-diyl dihexanoate; 2-acetamido-2-deoxy-beta-D-glucopyranose	2018	3.30	6CUD	^92^
	TRPC4	Transient receptor potential cation channel subfamily c member 4a	Zebrafish	(2R)-3-(phosphonooxy)propane-1,2-diyl dihexanoate; 4-[4-[[4,4-bis(fluoranyl)cyclohexyl]methyl]-3-oxidanylidene-piperazin-1-yl]-5-chloranyl-1 ~ {H}-pyridazin-6-one; Calcium ion	2020	3.80	7B05	^655^
	TRPC5	Short transient receptor potential channel 5	Human	(2S)-3-(hexadecanoyloxy)-2-[(9Z)-octadec-9-enoyloxy]propyl 2-(trimethylammonio)ethyl phosphate; Phosphatidylethanolamine; (2S)-2-(hexadecanoyloxy)-3-hydroxypropyl (9Z)-octadec-9-enoate; Cholesterolhemisuccinate; Zino ion; Calcium ion	2021	3.00	7E4T	^656^
	TRPC6	Short transient receptor potential channel 6	Mouse	/	2018	3.80	6CV9	^90^
TRPV	TRPV1	Transient receptor potential cation channel subfamily V member 1	Rat	/	2013	3.27	3J5P	^75^
				[(2 ~ {R})-1-[2-azanylethoxy(oxidanyl)phosphoryl]oxy-3-hexadecanoyloxy-propan-2-yl] (~{Z})-octadec-9-enoate; 1-palmitoyl-2-oleoyl-sn-glycero-3-phosphocholine; Sodium ion; Phosphatidylinositol; 1-palmitoyl-2-oleoyl-sn-glycero-3-phosphoglycerol	2021	2.63	7LP9	^657^
		Transient receptor potential cation channel subfamily V member 1, Kappa-theraphotoxin-Cg1a 1	Rat, Chilobrachys guangxiensis	/	2013	3.8	3J5Q	^76^
	TRPV2	Transient receptor potential cation channel subfamily V member 2	Rat	/	2019	3.70	6U84	^658^
	TRPV3	Transient receptor potential cation channel subfamily V member 3	Mouse	(2 S)-3-(hexadecanoyloxy)-2-[(9Z)-octadec-9-enoyloxy]propyl 2-(trimethylammonio)ethyl phosphate; Diundecyl phosphatidyl choline	2020	3.30	6LGP	^659^
	TRPV4	Transient receptor potential cation channel subfamily V member 4	Human	Phosphate ion; Glycerol	2012	2.85	4DX1	^660^
	TRPV5	Transient receptor potential cation channel subfamily V member 5	Rabbit	/	2022	3.20	7T6J	^661^
	TRPV6	Transient receptor potential cation channel subfamily V member 6	Human	/	2017	3.60	6BO8	^662^
TRPA1		Transient receptor potential cation channel subfamily A member 1	Human	1-palmitoyl-2-oleoyl-sn-glycero-3-phosphocholine; [(2 ~ {R})-1-[2-azanylethoxy(oxidanyl)phosphoryl]oxy-3-hexadecanoyloxy-propan-2-yl] (~{Z})-octadec-9-enoate; N-benzylthioformamide	2020	3.06	6PQP	^663^
TRPM	TRPM2	Transient receptor potential cation channel subfamily M member 2	Human	/	2018	3.60	6MIX	^103^
	TRPM3	Transient receptor potential cation channel, subfamily M, member 3	Mouse	1,2-DIACYL-GLYCEROL-3-SN-PHOSPHATE; (3beta,14beta,17beta,25R)-3-[4-methoxy-3-(methoxymethyl)butoxy]spirost-5-en; Sodium ion	2022	3.20	8DDR	^664^
	TRPM4	Transient receptor potential cation channel subfamily M member 4	Mouse	Sodium ion	2017	3.14	6BCJ	^109^
	TRPM5	Transient receptor potential melastatin 5	Zebrafish	(2R)-2-(hydroxymethyl)-4-{[(25R)-10alpha,14beta,17beta-spirost-5-en-3beta-yl]oxy}butyl 4-O-alpha-D-glucopyranosyl-beta-D-glucopyranoside; (25R)-14beta,17beta-spirost-5-en-3beta-ol; 2-acetamido-2-deoxy-beta-D-glucopyranose	2021	2.80	7MBP	^665^
	TRPM7	TRPM7	Mouse	/	2018	4.10	6BWF	^666^
	TRPM8	Transient receptor potential cation channel subfamily M member 8	Mouse	Calcium ion	2022	2.88	7WRB	^667^
TRPN		No mechanoreceptor potential C isoform L	Drosophila melanogaster	1,2-DIACYL-SN-GLYCERO-3-PHOSHOCHOLINE	2017	3.55	5VKQ	^142^
TRPP	TRPP2	Polycystin-2	Human	2-acetamido-2-deoxy-beta-D-glucopyranose	2016	3.00	5T4D	^130^
	TRPP3	Polycystic kidney disease 2-like 1 protein	Human	2-acetamido-2-deoxy-beta-D-glucopyranose	2018	3.38	5Z1W	^136^
			Human	/	2012	2.80	4GIF	^126^
TRPML	TRPML1	Mucolipin-1	Mouse	Sodium ion	2022	2.60	7SQ8	^668^
	TRPML2	Mucolipin-2	Human	2-(N-MORPHOLINO)-ethanesulfonic acid; Glycerol; Potassium ion; Chloride ion	2019	2.00	6HRR	^669^
	TRPML3	Mucolipin-3	Callithrix jacchus	1,2-Distearoyl-sn-glycerophosphoethanolamine; CHOLESTEROL HEMISUCCINATE; Sodium ion	2017	2.94	5W3S	^115^

#### TRPVs

The earliest structural information obtained for the TRPV subfamily was the X-ray crystal structures of the isolated cytoplasmic ankyrin repeat domain from TRPV2 in 2006, immediately followed by similar structures for other TRPV channels.^70,71^ As is true for all TRP channels, TRPV channels are tetrameric and each monomer features the classic six transmembrane helix architecture of voltage-gated ion channels in its TMD.^72^ The overall sequence similarity of TRPVs is more than 40%. TRPV protein has an ~400–450-residue N-terminal domain with 3–6 intracellular ankyrin repeats, which are essential for channel function.^70,73^ An ~150-residue C-terminus that acts as a platform for interactions with other proteins and ligands.^74^

Liao et al. used advances in EM to determine the structure of the mammalian TRPV1 channel at a resolution of 3.4 Å.^75^ S1–S4 form a bundle similar to the voltage-sensing domain in voltage-sensitive ion channels.^72^ S5 and S6 extend from the S1–S4 bundle, and the conserved “TRP structural domain” interacts with the S4–S5 linker.^72,76^ TRPV1 has a wide extracellular “mouth” with short selective filtering.^75^ This domain-swapped pore domain consists of S5 and S6, which form the central pore and lower gate, whereas a short loop and helix between S5 and S6, termed the pore helix (PH), forms the upper gate or selectivity filter.^72^ The dynamic communication between the upper and lower gates may be the basis for the integration of various physiological signals.^76^ Notably, the motion of S6 in the TRPV1 structure can be roughly described as small, medium, or large. When the motion is small, the π-helix is retained, and the channel gate is formed by I679.^77^ In the intermediate category, the π-helix transitions to the α-helix, and the gate is formed by M682.^77^ When S5/S6 motion is large, the π-helix conformation is observed, and the open gate (9.8 Å) is formed by I679.^77^ However, transitions between π and α have also been observed in TRPV2 and V3 structures, but the functional consequences of such transitions remain to be fully understood.^78,79^

#### TRPCs

Given the great advancements in single-particle cryo-EM technology, high-resolution structures of TRPCs became available in 2018.^80^ TRPC2 is thought to be a pseudogene in humans.^81^ The overall architecture of the homologous TRPC3, TRPC4, TRPC5, and TRPC6 is consistent with that of TRP.^82–84^ The structure of TRPCs is similar to the voltage sensor structural domains of voltage-gated K^+^, Na^+^ and Ca^2+^ channels, with S5–S6 forming a structurally conserved ion conductance or pore domain common to all TRP channels, voltage-gated channels, inwardly-rectifying K channels, and bacterial K and Na channels.^85,86^ All TRPC structures contain a three-helix region designated as the pre-S1 elbow and pre-S1 helix before the transmembrane S1 helix.^87,88^ This region is halfway embedded in the membrane, with its N-terminal exposed to the cytoplasmic side to connect with a long stretch of tightly folded linker helices located at the proximal N-terminus of each protomer.^89^ Immediately before the linker helices are the four ankyrin-like repeats that form the outskirt of the cytoplasmic architecture, which completely surrounds the four helical bundles composed of the second C-terminal helix.^90^

Currently, the resolved region of the cytoplasmic structural domain of TRPC4 reaches approximately 80 Å, which is relatively short compared with that of other TRP channels.^91^ Helices S5 and S6 are exchanged with the respective helix of the adjacent protomer and form a pore at the center of the quaternary channel with a negatively charged extracellular opening; residue E555 at the top end of the pore tower is conserved in TRPC1, TRPC4 and TRPC5 and forms a salt bridge with R556 of the adjacent protomer.^91^ Fan et al.^92^ proposed that TRPC3 is in a lipid-occupied closed state with a structure of 3.3 Å. TRPC3 has four elbow-like membrane reflux helices before the first transmembrane helix.^92^ The third transmembrane helix, S3, is very long, forms a unique transmembrane structural domain and constitutes an extracellular structural domain that may act as a sensor for external stimuli.^92^

#### TRPA

The *TRPA1* gene encodes a large protein consisting of about 1100 aa (1119 aa in humans, 1125 aa in rats, 1115 aa in mice, 1120 aa in zebrafish, 1197 aa in *Drosophila*, and 1193 aa in *C. elegans*), with an estimated molecular mass between 120 and 130 kDa.^93^ TRPA1 has distinctively large intracellular NH2 and COOH termini, which together account for about 80% of its molecular mass.^94^ Each subunit of TRPA1 consists of six transmembrane alpha sheets (S1–S6) plus a re-entrant pore loop between S5 and S6; homotetramers are formed by “domain exchange” interactions and establish a conserved transmembrane core.^95,96^ The distinguishing feature of TRPA1 is its long N-terminus with 14–18 predicted ankyrin repeats, each consisting of 33 aa.^97,98^ A structural domain containing a sequence of five ankyrin repeats surrounds the coiled coil and connects to another extended feature, which forms a crescent-shaped element.^95^ TRPA1 also possesses a distinct tetrameric coiled coil at the center of the channel, beneath the permeation pore. This stalk-like structure is stabilized by the interaction of positively charged residues on the exterior surface of the coiled coil with inositol polyphosphates.^94^ These interactions are essential for TRPA1 channel activity.^94,99,100^

#### TRPMs

In late 2017, several TRPM structures were solved using single-particle cryo-EM.^101^ TRPMs have four shared homologous regions: a TMD consisting of six transmembrane helices, a TRP helix, and C-terminal domains containing the conserved TRP domain followed by a coiled-coil region that differs among members.^16,102^ Despite their common structural features, the members of the TRPM subfamily are less conserved than those of other subfamilies. Several TRPM channels, such as TRPM2, uniquely contain a functional nucleoside diphosphate linked to another moiety/ADP ribose domain and a kinase domain that resembles to protein kinase A to a certain extent.^103,104^ TRPM2 is structurally characterized by its C-terminal NUDT9-H structural domain, a homologue of the human ADP-ribose pyrophosphatase NUDT912.^105^ In other words, these TRPMs combine features of ion channels and enzymes and are thus referred to by some as “chanzymes”.^106–108^ The ion conductance pore of TRPM2 is restricted at two locations, a selective filter located close to the extracellular entrance, which is formed by a hinge connecting the PH and the P ring, and a gate lined by S6 near the inner end of the cell.^105^

TRPM4 consists of three layers, namely, the N-terminal nucleotide-binding domain, ankyrin repeat domain, and the C-terminal coiled-coil helix, which form the base layer of TRPM4.^109^ The middle layer consists of a linking helix structure containing 12 helices and forming a scaffold for multiple interactions between structures within the subunit.^109^ The S1–S6 and TRP structural domains embedded in lipid form the top layer of the channel.^109^ The C-end coiled spiral structure forms a parallel disc-shaped coil.^110^ The discoid coil is surrounded by a large, intertwined cytoplasmic structural domain consisting of four N-terminal TRPM homology regions (MHR1–4), which are highly conserved in the TRPM subfamily.^95,102,111^ Of these, MHR1–2 contain an eight-stranded β-sheet surrounded by eight α-sheets.^110^ By contrast, MHR3–4 are composed of stacked α-sheets and connected to the transmembrane structural domain via MHR4, which clasps the TRP structural domain; as a result, an interaction occurs between the cytoplasmic structural domain and the transmembrane core.^109,112^ In 2018, Autzen et al.^112^ compared the structures of Ca^2+^-free and Ca^2+^-bound TRPM4 and observed an additional density in the hydrophilic pocket on the cytoplasmic side of the S1–S4 structural domain (this density represents a true bound Ca^2+^).

The mouse TRPM8 channel is a three-layer homologous structure. The top transmembrane channel region consists of the pre-S1 structural domain, TMD, and TRP structural domain.^113^ The TMD consists of a voltage-sensor-like structure (VSLD) consisting of transmembrane helices S1 to S4, and a pore domain formed by S5 and S6 helices,^113,114^ similar to the previously identified TRPV structure.^75,79^ The TMD of TRPM8 also has several features that distinguish it from the structure of other TRP ion channels. First, relative to the structure of apo TRPV1, the PH of TRPM8_FA_ is positioned 12 Å away from the central axis, tilted by 8°, and shifted by 5 Å toward the outside of the cell.^101^ Second, no non-α helical elements (e.g., 310 or π helices) can be found in the TMs of TRPM8, which in other TRP channels are proposed to provide helical bending points for channel gating.^115^ Finally, although TRPV channels have a pre-cellular S1 helix, TRPM8_FA_ contains three additional helices between S1 and the presumed pre-cellular S1 helix in the membrane region.^101^ Diver et al.^116^ observed a substantial rearrangement in the S4–S5 linker in the TRPM8 structure, and it repositioned the S1–S4 and pore domains relative to the TRP helix.

#### TRPPs

The TRPP subfamily has three members (TRPP2, TRPP3, and TRPP5).^117^ The PKD1 and PKD2 genes encoding polycystin-1 and polycystin-2 proteins, respectively, were originally named TRPP1 and TRPP2.^118–120^ However, members of the PKD1 family do not belong to the TRP family. PKD proteins are divided into two groups based on sequence homology: typical isoforms with 6 TMs (PKD2 subfamily), such as TRPP2, TRPP3, and TRPP5; and another group with 11 TMs and a short intracellular C-terminal tail (PKD1 subfamily), represented by PKD1, PKD1L1, PKD1L2, PKD1L3 and PKD-REJ7.^121^ The TRPP protein can form homotetramers and heterotetramers,^122,123^ such as the PKD1/TRPP2 and PKD1L3/TRPP3 complexes, with members of the PKD1 family.^124–126^

PKD1 is a 4,303-aa, 465-kDa integral membrane protein containing a large N-terminal extracellular structural domain and an intracellular C-terminal structural domain.^127^ Unlike PKD1, TRPP2 is a 968-aa, 110-kDa protein with 6 TMs and a pore-forming loop,^128^ and has intracellular amino and carboxyl termini.^129^ Shen et al.^130^ showed that TRPP2 is a Na/K conducting channel with low permeability and small single-channel conductance to Ca^2+^. The complexes of PKD1 and TRPP2 exhibit a 1:3 structural ratio.^131^ TRPP2 and PKD1 bind directly through their C-termini to form a complex containing three TRPP2 and one PKD1.^132^ This association involves a coiled-coil structure at the C-terminus of both proteins.^125^ R807X, E837X, and R872X of TRPP2 and R4227X and Y4236X of PKD1 can lead to deletion of the coiled-coil structural domain, which results in the mutation of ADPKD.^133,134^

TRPP3 (polycystin-2 like 1 (PKD2L1)) and TRPP2 share high sequence similarity (79% homology and 62% identity).^135^ Unlike TRPP2, the PH and S6 of TRPP3 are involved in the opening of the upper and lower gates to adopt an open conformation.^136^ Su et al.^136^ suggested that the simultaneous occurrence of gate opening and voltage-sensing domain (VSD) conformational changes in TRPP3 is caused by coupling between the VSD and pore domains. PKD1L3/TRPP3 complexes form calcium-permeable, non-selective cation channels.^137,138^ The structure of PKD1L3/TRPP3 in the apo and Ca^2+^-loaded states shows two Ca^2+^ binding sites at selectivity filter and VSD_III_ respectively.^139^ It also has a 1:3 stoichiometry TRP structure protected by Lys2069 of PKD1L3 and Asp523 from the three subunits of TRPP3.^139^

#### TRPN and TRPMLs

*Drosophila* NOMPC protein is the first member of the TRPN subfamily.^140^ The *Drosophila* NOMPC protein shares significant homology with the TRP superfamily.^52^ The N-terminus of NOMPC contains 29 ankyrin repeats, the largest number among TRP channels.^141^ Unlike other TRP family proteins, the ankyrin repeat structure of NOMPC is a quadruple structure, with each structural domain acting like a helical spring.^142^ The TMD of each NOMPC subunit consists of six transmembrane α-helices (S1–S6) and a re-entrant pore loop located between S5 and S6.^142^ The TRP structural domain is sandwiched between the linker helix and the S4–S5 linker. The short helix following the TRP structural domain is wrapped around the elbow in front of S2 and S1.^142^ However, the C-terminus is mostly unstructured, with a short helix fragment interacting with the linker domain.^142^ No TRPN has been found in mammals.

The TRPML subfamily is defined by a human protein, mucolipin 1.^143^ Mucolipin 1 (TRPML1) is a 580 aa-long protein probably restricted to intracellular vesicles.^144^ TRPML1 contains two proline-rich regions, namely, a lipase serine active site in the N-terminus and a dileucine motif suggestive of lysosomal targeting in the C-terminus.^64^ TRPML1 is characterized by its longer S2 helix than that of PKD2 and protrudes from the membrane bilayer-embedded portion of the cell membrane, with an extension of the N-terminal S1 helix (pre-S1) that includes two small alpha helices (α1 and α2) rich in many basic aa.^145^ Several aromatic and hydrophobic residues in the PH 1, S5, and S6 helices of TRPML1 and the S6 helix of the adjacent subunit form a hydrophobic cavity to accommodate the agonist.^145^ Phosphatidylinositol-3,5-bisphosphate binds to the N-terminal end of TRPML1, that is away from the pore, and a helix-turn-helix extension between the S2 and S3 segments may link ligand binding to pore opening.^146^ Two other members of the TRPML subfamily, namely, TRPML2 and TRPML3, are encoded by *MCOLN2* and *MCOLN3* genes, respectively.^147^ Similar to TRPML1, they are active in late endosomes and lysosomes.^148^ TRPML3 is divided into an extracellular structural domain (ECD), a TMD, and a cytosolic structural domain.^115^ The ECD consists of two β-sheets and two extracellular helices that form a ring that caps the extracellular side of the channel and is structurally similar to the ECD of TRPML1 and PKD2.^115,130,149,150^ Unlike PKD2, in which the ECD interacts extensively with the TMD, in TRPML3, minimal interaction exists between the ECD and the TMD.^115,151^

### Agonists and antagonists

Various stimuli, including noxious and innocuous heat or cold stimuli, changes in osmolarity, proton gradients, and irritant compounds, have the potential to activate TRP channels.^152^ The activity of TRP channels is also modulated by several natural products, herbs, and compounds of plant origin. For stimulation by temperature, each temperature-sensing TRP channel has a specific activation threshold. Notably, numerous TRP agonists and antagonists are not specific; cross-reactivity can be observed between different TRP channels.

Several potent small-molecule agonists and inhibitors of TRPV1, TRPV4, and TRPA1 have entered clinical trials for the treatment of inflammatory, neuropathic, and visceral pain; however, the therapeutic mechanism of action of these compounds is unclear.^153^ The TRPA1 agonist cinnamaldehyde improves the capability of glucose metabolism by slowing down the gastric emptying rate and food intake via the TRPA1-ghrelin pathway.^154^ In addition, oral administration of HC-030031, an inhibitor of TRPA1, significantly reversed mechanical hypersensitivity in a spinal nerve ligation model of complete Freund’s adjuvant (CFA)-induced inflammatory pain and neuropathic pain in rats.^155^ However, the mechanism by which it regulates TRPA1 is unclear.

Capsaicin is the most classic TRPV1 agonist, and it has a role in enhancing pain.^156^ Capsaicin has a “tail up, head down” conformation. Molecular dynamic simulations revealed that the capsaicin molecule flips from extracellular to intracellular and can subsequently enter the intracellular TRPV1 binding site.^157^ Oral administration of GSK1016790A activates TRPV4 and reduces atherosclerotic plaque formation.^158^

Menthol is known to be an agonist of TRPM8. However, the mechanism by which TRPM8 channels respond to this drug is not well understood. Mutations in residues within the transmembrane and TRP structural domains impair the efficacy of menthol, such as Y745, R842, and L1009 (all residue numbers belong to mouse TRPM8), impair the efficacy of menthol, which suggests that this structural domain affects initial binding downstream.^159^ Later, in a cryo-EM of the structure of full-length avian TRPM8, the menthol binding site was located within a voltage-sensor-like structural domain.^101^ The cavity near Y745 and R842 was identified as the binding pocket for menthol. Recently, Xu et al. proposed that menthol, with its hydroxyl group as a hand, specifically grasps R842, and with its isopropyl group as a leg, it stands on I846 and L843, combining TRPM8 by a “grasp and stand” mechanism.^160^ Thus, numerous agonists and antagonists of TRPs have been reported (Table 3).

**Table 3:** Agonists and inhibitors of TRP channels

TRP channel	Agonists	Inhibitors	Applications	Ref.
TRPA1	Cinnamaldehyde, Sanguinarine, Probenecid, JT010, Methylglyoxal	GDC-0334, HC-030031, Chembridge-5861528, GRC17536, ODM-108, A-967079	Pain, inflammation, asthma, cancer, obesity, cough	^38^
TRPV1	Capsaicin, Resiniferatoxin, Oxytocin, Rinvanil, Arvanil	SB-705498, AMG517, JNJ17203212, AZD1386	Pain, cough, metabolism, epilepsy	^238^
TRPV2	O1821	Tranilast, piperone	Alzheimer’s disease, rheumatoid arthritis, cardiomyopathy	^670–672^
TRPV3	Carvacrol, camphor, incensole acetate, serratol, menthol,	Coumarin, osthole, verbascoside, pulchranin A, ruthenium red, PC5, lidocaine, 17R-RvD1	Skin disorders, anxiety, convulsions, pain, itch	^673,674^
TRPV4	4α-PDD, GSK1016790A, RN-1747	GSK2798745, HC-067047, GSK2193874, PF-05214030	Edema, inflammation	^675^
TRPM8	Menthol, Rotundifolone, Eucalyptol, Cubebol	PF-05105679, Cannabidivarin, AMG-333, SML0893	Vasoconstriction, blood glucose regulation, dry eyes, pain	^646^
TRPM7	Clozapine, Naltriben	Ruthenium red, 2-APB, SKF-96365, Ginsenoside Rg3	Cancer, cardiac fibrosis	^676^
TRPM3	CIM0216, PregS, Nifedipine	Promethazine	Pain, chronic fatigue syndrome	^271,677^
TRPM2	NAD, NAADP	8-Br-cADPR, Flufenamic acid, Imidazole anti-fungal agents, 2-APB	Insulin release, cytokine production, endothelial permeability	^678^
TRPC3	GSK1702934A, PLC	1-benzilpiperadine, Pyr3, 2-APB	Hind-limb ischemia, myocardial ischaemia, hypertension, convulsions	^679–681^
TRPC6	3-(3-,4-Dihydro-6,7-dimethoxy-3,3-dimethyl-1-isoquinolinyl)-2H-1-benzopyran-2-one, oxygen-glucose deprivation	SKF96365, Pyrazolo[1,5-a]pyrimidine, larixyl acetate, SH045	Alzheimer’s disease, renal ischemic reperfusion injury, gastric cancer, endothelial dysfunction	^682–684^
TRPML	MK6-83, ML-SA1	Trans-ML-SI3, ML-SI1, ML-SI3	Glioblastoma, antiviral, schistosomiasis	^685–687^

*NAADP* nicotinic acid-adenine dinucleotide phosphate, *NAD* nicotinamide adenine dinucleotide

## Signaling pathways affected by TRP channels

TRP channels exhibit ion selectivity. For example, TRPV5 and TRPV6 are selective toward Ca^2+^ and have a selective filter similar to that of voltage-gated potassium channels.^161^ Monovalent, divalent, and large organic cations pass through TRPV1, V2, V3, and TRPA1 and may allow dynamic ion selectivity.^77^ TRPM3 is found to be a constitutive Ca^2+^ and Mn^2+^ permeable channel, as shown by in vitro studies.^162^ TRPM4 and TRPM5 are voltage-regulated and conduct monovalent cations, such as Na^+^ or K^+^, without significant Ca^2+^ penetration.^163^ The activity of TRPM7 is regulated by intracellular Ca^2+^ and Mg^2+^ and is permeable to almost all physiological divalent cations and trace metals such as Ni^2+^.^164^ TRPM6 is also permeable to trace metals, including Mg^2+^ and Ca^2+^, and is inhibited by intracellular Mg^2+^.^165^ Activation of TRP channels causes ionic changes inside and outside the cell to trigger downstream pathways. Multiple signaling pathways are affected by TRPs, and these pathways include the mitogen-activated protein kinase (MAPK) pathway, transforming growth factor (TGF)-β signaling pathway, nuclear factor kappa-B (NF-κB) pathway, and AMP-activated protein kinase (AMPK) pathway.

### MAPK pathway

The MAPK pathway includes extracellular signal-regulated kinases (ERK MAPK), c-Jun N-terminal kinases (JNK) or stress-activated protein kinases, and p38 MAPK.^166^ MAPK pathways regulate processes ranging from proliferation and differentiation to apoptosis.^167^ They affect gene expression, metabolism, cell division, cell morphology, and cell survival.^168^

In most cases, inflammatory stimulation of cells causes an inward flow of calcium.^169^ Several members of TRP channels act as calcium-permeable channels, and their activation induces calcium influx, which is involved in the regulation of the MAPK signaling pathway.^170^ In human corneal epithelial cells, the activation of TRPV1 receptor was followed by an increase in Ca^2+^ influx, which led to MAPK activation, followed by the increased release of interleukin (IL)-6 and IL-8, inducing an inflammatory response.^171^ Upregulated TRPV5 expression was observed in chondrocytes from rats with osteoarthritis, which regulated a Ca^2+^ influx to activate the phosphorylation of calmodulin-dependent kinase II (CaMKII).^172^ Activated p-CaMKII causes extracellular signaling-regulating phosphorylation of ERK 1/2, JNK, and p38 and plays a key role in chondrocyte apoptosis.^172^ Furthermore, activation of TRPM8 in human bronchial epithelial cells promotes Ca^2+^ influx, which subsequently leads to increased expressions of IL-6, IL-8, and tumor necrosis factor (TNF)-α via the upregulation of p-ERK and activation of NF-κB, which amplify inflammation.^173^

Activation of the ERK1/2 pathway in neurons may be involved in acute visceral pain.^174^ This activation is induced by TRPA1.^175^ Experimental results by Kondo et al. suggested that mechanical stimulation by TRPA1 may generate action potentials that lead to phosphorylation of ERK1/2 and p38 in dorsal root ganglion (DRG) neurons, which are further involved in the development of visceral pain.^176^ Activation of TRPV4 regulates the MAPK and phosphatidylinositol 3-kinase (PI3K)/protein kinase B (Akt) signaling pathways, which regulate cell death and survival.^177^ When TRPV4 channels are activated, the protein levels of p-p38 MAPK are significantly increase, which induces the apoptosis, which is associated with cerebral ischemic injury.^178^

TRP channel regulation of the MAPK signaling pathway not only affects pain and inflammation but may also be involved in the fibrotic process. Inhibition of TRPM7 expression reduces liver fibrosis by suppressing the activation and proliferation of hepatic stellate cells, a process that possibly involves the MAPK signaling pathway.^179^ In addition, TRPV4-mediated Ca^2+^ influx is involved in regulating the differentiation of human ventricular cardiac fibroblasts to myofibroblasts via the MAPK/ERK pathway.^180^

### TGF-β signaling pathway

The TGF-β superfamily consists of TGF-β1-3, activins and inhibins, growth differentiation factors, myostatin, and BMPs.^181^ Dysregulation of TGF-β family signaling can lead to a plethora of developmental disorders and diseases, including cancer, immune dysfunction, and fibrosis.^182,183^

TGF-β signaling underlies cardiac fibrosis.^184^ Activation of TRPV3 was observed in stress-overloaded rats, and it upregulated protein expressions of collagen I, collagen III, TGF-β1, cyclin E, and cell cycle protein-dependent kinase 2 (CDK2).^185^ Meanwhile, blocking the TGF-β1 pathway partially reversed the effect of TRPV3 activation.^185^ This finding suggests that TRPV3 activation exacerbated fibrosis in pressure-overloaded rat hearts by promoting the proliferation of cardiac fibroblasts through the TGF-β1/CDK2/cyclin E pathway.^185^

Changes in TRPC3 expression and atrial fibrosis are closely related.^186^ Angiotensin (Ang) II induces migration and proliferation of atrial fibroblasts and upregulates the expression levels of TRPC3 and fibrosis biomarkers.^187^ The TRPC3-selective blocker Pyr3 significantly attenuated Ang II-induced upregulation of TRPC3, collagen I, collagen III, and TGF-β1 through the molecular mechanism of the TGF-β/Smad2/3 signaling pathway and attenuates neonatal rat atrial fibroblast migration and proliferation.^187^

TRPV1 exerts protective effect against cardiac and renal fibrosis, mainly through the TGF-β signaling pathway.^188^ TRPV1 was significantly downregulated in human liver fibrotic tissues.^189^ By contrast, knockdown of *TRPV1* resulted in a significant increase in the expression of various liver fibrosis markers, and enhanced the promotion of TGF-β in hematopoietic stem cell proliferation, cell cycle, apoptosis and extracellular matrix expression.^189^ Selective activation of TRPV1 in mice with renal fibrosis inhibited α-smooth muscle actin (SMA) but promoted E-cadherin expression in human proximal tubular epithelial cells primarily through the inhibition of TGF-β1-Smad2/3 signaling.^190^ These data suggest that TRPV1 activation attenuates the progression of cardiac and renal fibrosis by inhibiting TGF-β and its downstream regulatory signaling pathways,^191^ and exerts cardioprotective and renoprotective effects.

### Canonical NF-κB pathway

The canonical NF-κB is activated by various stimuli to transduce rapid but transient transcriptional activity to regulate the expressions of various proinflammatory genes and also serves as a critical mediator of the inflammatory response.^192^

Kang et al.^193^ observed that by blocking TRPA1 induced a decrease in IL-6 and IL-13 levels were induced. Furthermore, blockade of NF-κB inhibited TRPA1 expression, whereas blockade of TRPA1 had no significant effect on NF-κB activation.^193^ However, several researchers have come to the opposite conclusion. Lee et al.^194^ concluded that inhibition of TRPA1 channel activity inhibited the production of pro-inflammatory cytokines and the activity of NF-κB.

In a model of cisplatin-induced nephrotoxicity, blockade of TRPA1 alleviated apoptosis, reduced the levels of IL-1β, IL-6, TNF-α, and interferon (IFN)-γ, decreased the levels of cleaved caspase-3, cleaved poly (ADP-ribose) polymerase PARP, and inducible nitric oxide synthase (iNOS), reduced the expression of p-IKKβ, p-JNK, p-ERK and p-p38, and enhanced the expression of IκBα.^195^ TRPA1 expression was increased in renal tubular epithelial cells from patients with acute kidney injury. Activation of TRPA1 increased intracellular Ca^2+^ levels, increased NADPH oxidase activity, activated MAPK/NF-κB signaling, and increased IL-8 levels.^196^ In conclusion, TRPA1 regulates inflammation through the MAPK/NF-κB signaling pathway.

TRPV4 is involved in the molecular mechanisms of inflammation mainly through NF-κB. In a mouse model of temporal lobe epilepsy, activation of TRPV4 increased Toll-like receptor 4 (TLR4) expression, which led to the phosphorylation of IκK and IκBα; as a result, NF-κB activation and nuclear translocation occurred, with a resulting increase in the levels of pro-inflammatory cytokines.^197^ Thus, blocking TRPV4 downregulates high mobility group box 1 (HMGB1)/TLR4/IκK/κBα/NF-κB signaling, which produces an anti-inflammatory response and neuroprotective capacity.^197^

Scholars have proposed the involvement of TRPM8 channels in the DRG, through the regulation of NF-κB signaling, in the pathogenesis of hyperalgesia in neuropathic pain rats.^198^ Activation of TRPM8, which inhibits nuclear localization of NF-κB, led to the repression of *TNF* (encoding TNF-α) gene transcription.^199^

TRPC6 is beneficial in alleviating inflammation and apoptosis. Activated TRPC6 reduces Ca^2+^ entry into cells, which inhibits nuclear translocation and phosphorylation of NF-κB and reduces apoptosis, cytotoxicity, and inflammatory responses.^200^ By contrast, TRPC1 channels cause a Ca^2+^ influx downstream of Ca^2+^/calmodulin-dependent protein kinase β (CaMKKβ), which promotes NF-κB activation.^201^

### AMPK pathway

The AMPK pathway promotes mitochondrial health and homeostasis and plays a major role in the regulation of cellular energy homeostasis.^202^ Inhibition of TRPV1 attenuates rutaecarpine-induced intracellular Ca^2+^ levels and phosphorylation of CaMKII, CaMKKβ, AMPK, and endothelial NO synthase.^203^ In addition, increased expression of TRPV1 in patients with blistering skin injury led to an upregulation of intracellular Ca^2+^ levels and CaMKKβ, AMPK, unc-51-like kinase 1 and mammalian target of rapamycin (mTOR) activities, which cause skin toxicity through excessive activation of autophagy.^204^

Upregulation of TRPC5 protein expression levels mediates cell survival autophagy via the CaMKKβ/AMPKα/mTOR pathway.^205^ Notably, not all TRP channels regulate the AMPK signaling pathway. By contrast, activation of AMPK normalizes TRPC6 expression.^206^

Altogether, these results suggest that TRPs can perform important biological functions by regulating downstream signaling pathways to influence disease development. Targeting key molecules and pathways that mediate abnormal states may be critical for the management of TRP channel-related diseases.

## Physiological functions of TRP channels

TRPs have long been an important topic in the scientific community, with a growing number of publications suggesting their involvement in various of biological processes. TRPs are distributed in a variety of tissues, and have a wide range of physiological functions.^207^ Thus, the biological functions of TRPs must be explored to further understand the development and progression of diseases.

### Pain

Pain is the primary reason why people seek medical care.^208^ Until the 1960s, pain was considered an inevitable sensory response to tissue damage.^209^ Pain can be categorized as nociceptive (from tissue injury), neuropathic (from nerve injury), or nociplastic (from a sensitized nervous system), all of which affect work-up and treatment decisions at every level.^210^ Opioids are effective pain relievers, but acting on the brain, they can become addictive.^211^ Pain exerts an enormous personal and economic burden.^208,212^ A rational strategy to avoid the side effects of opioid is to target the starting point of the pain pathway: the neuroreceptors that produce pain.^213^ Certain members of the TRP family (TRPV, TRPA, and TRPM) constitute key detectors and transducers of nociceptive stimuli.^214^ Therefore, researchers have expressed great interest in the analysis of TRP channels.

#### Activation of TRPA1 induces pain

TRPA1 plays a role in various forms of pain, including neuropathic cold pain, inflammatory pain, and hereditary episodic pain syndromes.^215,216^ As shown by dermal experiments, the activation of TRPA1 by certain compounds triggers pain and heat sensations.^215–217^ Sustained activation of TRPA1 by endogenous substances induces chronic pain involved in various disorders,^218^ including fibromyalgia, osteoarthritis, inflammation, diabetes, obesity, migraine, neuropathy, bronchitis, and emphysema.^219^

**Fig. 4:**
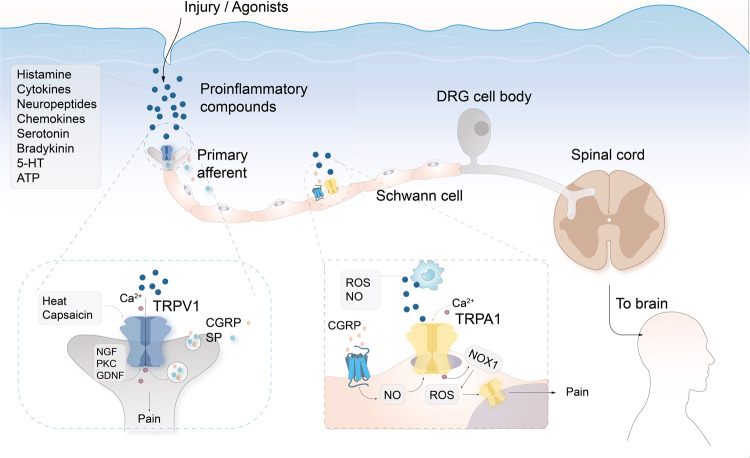
TRPA1 and TRPV1 in pain regulation. Sensory nervous system injury, heat, or agonist activation of TRPV1, leading to Ca^2+^ influx and release of SP and CGRP. At this point, the nerve fibers release a variety of trophic factors (NGF, and glial cell-derived neurotrophic factor (GDNF)) and PKC to reduce the sensitivity of TRPV1. This condition puts the nerve fibers and neurons in an excitable state, which causes ectopic discharges and leading to pain. Following tissue injury, pro-inflammatory substances are released, including cytokines, chemokines and more, which causes the recruitment of activated macrophages to the lesion site. Macrophage-dependent ROS and NO evoke Ca^2+^/NADPH oxidase 1 (NOX1), respectively, and subsequently activate TRPA1 in phagocytosed neuroreceptors to maintain mechanical nociception. On the other hand, CGRP activates CGPR receptors on Schwann cells. CGPR receptors are transported to the endosomes and sustained signaling leads to the release of nitric oxide. Nitric oxide targets TRPA1 in Schwann cells, which excites neurons and transmits pain signals. CGRP calcitonin gene-related peptide, SP substance P, NGF nerve growth factor, GDNF glial cell-derived neurotrophic factor, PKC protein kinase C, ROS reactive oxygen species, NO nitric oxide, NOX1 NADPH oxidase 1

Chembridge-5861528 blocks TRPA1 and relieves mechanical nociception after nerve injury.^220^ Koh et al.^221^ observed that inhibition of TRPA1 expression reduced the development of mechanical, thermal, and cold heterosensitivity. The experimental results of Sagalajev et al.^222^ showed that injury-induced oxidative stress in the amygdala stimulated TRPA1 to promote neuropathic pain behavior. This pain perception involved the inhibition of medullary serotonergic feedback inhibition acting on spinal 5-HT1A receptors, which was reversed by TRPA1 antagonists.^223,224^ In Schwann cells, activation of TRPA1 induces NADPH oxidase 1-dependent H_2_O_2_ release, maintains macrophage infiltration of injured nerves, and sends paracrine signals to activate TRPA1 in phagocytosed nociceptors to maintain mechanical nociception (Fig. 4).^225^

Activation of TRPA1 channels leads to an influx of sodium and calcium ions, which induces the depolarization of nociceptive nerve ending that is needed to generate the centrally propagating nociceptive signal.^226^ TRPA1 is expressed not only on peripheral but also on central terminals of nociceptive primary afferent nerve fibers.^227^ On central terminals located within the spinal dorsal horn, TRPA1 amplifies glutamate release and thereby transmission of nociceptive signals to spinal interneurons and projection neurons.^228,229^

Topical administration of selective TRPA1 agonists in vivo induced nociceptive behavior in experimental animals and pain sensation in human beings.^216,230^ Bautista et al.^231^ used TRPA1-deficient mice to better demonstrate that environmental stimulants and endogenous analgesics depolarize nociceptive receptors via TRPA1 to elicit inflammatory pain. TRPA1 antagonists showed no effect on all pain. Intrathecal application of a low dose of TRPA1 antagonist attenuated formalin-induced tactile allodynia but had no effect on acute pain behavior.^232^ In addition, systemic administration of HC-030031 (TRPA1 antagonist) showed to have anti-isokinetic and inflammatory pain efficacy in a CFA model.^155^ Interestingly, low doses of intrathecal HC-030031 can also able to attenuate pain.^233^ By contrast, a similar dose of a TRPA1 antagonist had no effect on healthy rats.^232,234^

#### TRPVs in pain

TRPV1 is a heat-activated cation channel modulated by inflammatory mediators and contributes to acute and chronic pain.^235^ About 30% of the TRPA1-expressing neurons co-express TRPV1.^93^ TRPV1, which is widely expressed in bipolar neurons, transmits sensory information from the periphery to the somatosensory cortex via the spinal cord.^236,237^ Stimulation of TRPV1 evokes a burning sensation, which reflects the central role of the channel in pain.^238^

*Trpv1* knockout mice in an inflammatory model exhibited attenuated thermal nociception, which sparked great interest among researchers to develop TRPV1 antagonists with analgesic properties.^239,240^ TRPV1 is involved in toxic chemicals and heat-induced pain and promotes peripheral sensitization.^241^ Analgesics may enhance nociceptive receptor activity by increasing TRPV1 expression.^242^

In the case of cutaneous and visceral pain, the downregulation of TRPV1 activity produces antinociceptive effects.^243,244^ TRPV1 in skin non-neuronal cells may release pro-inflammatory agents acting on nociceptive endings, which sensitizes neuron-expressed TRPV1 channels and triggers pain signals.^245,246^

TRPV4 primarily mediates inflammatory pain and neuropathic pain. Colonic mechanical pain and nociception were significantly relieved in *Trpv4* knockout mice.^247^ In mice, topical treatment with a TRPV4-selective inhibitor decreased UVB-evoked pain behavior, epidermal tissue damage, and endothelin-1 expression.^248^ In sunburned humans, elevated expressions of epidermal TRPV4 and endothelin-1 were observed.^249^ Activation of TRPV4 induced the transcriptional regulation and release of cytokines and tachykinins (substance P (SP) and calcitonin gene-related peptides (CGRP)),^247^ which ultimately led to chronic pain.^250^ Elevated prostaglandin E_2_ (PGE2) is also thought to contribute to TRPV4-mediated nociceptive behavior,^251^ which is important in pathological pain states associated with multiple diseases.^252^ In addition, TRPV4 plays a role in chronic DRG compression-induced neuropathic mechanical pain via the p38 MAPK pathway.^253^

Although studies on TRPVs related to pain have mainly focused on TRPV1 and TRPV4. TRPV2 and TRPV3 are also promising targets. TRPV2 is widely expressed in neuronal and non-neuronal cells.^254,255^ Increased expression of TRPV2 is associated with thermal hyperalgesia and mechanical thermal after oral mucosal incision.^256^ However, the role of TRPV2 in sensory neurons is currently unknown, and a limited number of studies have been conducted in relation to pain.

By contrast, TRPV3 does not act as extensively as other channels, and its studies have focused on skin diseases.^257^ TRPV3 has been suggested as a candidate transducer contributing to pain hypersensitivity associated with inflammatory states.^258,259^ Analgesic effects were observed in a model of pruritus treated with selective and potent inhibitors of the TRPV3 channel by Han et al.^260^. TRPVs are a promising therapeutic target in pain research.

#### TRPMs in pain

Numerous studies have linked the TRPM8 channel to pain. The channel is mainly expressed on peripheral sensory neurons and is known as a cold temperature sensor. However, it is also expressed in deep viscera. The administration of TRPM8 antagonists, knockdown of the *TRPM8* gene, or ablation of primary afferent neurons expressing TRPM8 can be effectively relieve the cold pain associated with tissue and nerve damage.^261,262^ The experimental results of Zuo et al.^263^ demonstrated that TRPM8 played a role in cold soreness and hyperalgesia following chronic trigeminal nerve injury. Activation of TRPM8 effectively reduces the painful behavior caused by chemical irritation, toxic heat, and inflammation.^264,265^ TRPM8 can also amplify the analgesic effects of their agonists by forming complexes with 5-HT-1B receptors.^266^ In addition, when TRPM8 od TRPA1 and TRPV1 are co-activated, an analgesic effect is produced.^267^

In addition to TRPM8, significant advances have been made in the role of TRPM2 in pain. Exposure of TRPM2 to H_2_O_2_ significantly increased mRNA and protein levels of pro-inflammatory cytokines (IL-1β, IL-6, and TNF-α) and a chemokine (CXCL2).^268,269^ Remarkably, the inhibition of TRPM2 expression can affect the elevated levels of cytokines and chemokines.^269^ TRPM2 may play a role in inflammatory pain. In animal experiments, TRPM2-deficient mice were unresponsive to mechanical and thermal nociception in a model of keratin-induced inflammation and exhibited reduced edema.^270^ Therefore, TRPM2 contributes to inflammatory pain by promoting peripheral immune functions.

TRPM2 and TRPM8 are the most relevant members of the family of TRPMs regarding pain, but the role of other members of the family in pain should not be underestimated. Pharmacological and genetic studies have shown that TRPM3 is associated with various pain patterns. Su et al.^271^ concluded that TRPM3 plays a role in thermal hyperalgesia and spontaneous pain but not in cold and mechanical hyperalgesia. The heat-activated TRPM3 ion channel is a potential target for new analgesics, but its mechanism in relation to pain is unknown and needs to be explored.

TRPs are arguably the most widely studied analgesic targets today. TRPV1 has been validated as a target for analgesic drugs in preclinical studies,^272^ and its side effects should not be underestimated. Whether other pain-related TRP channels act as the sole mediators of nociception, and the mechanisms of analgesia remains to be determined.

### IBD and complications

IBD, which is one of the global disorders of the 21st century,^273^ is a chronic recurrent disease of the gastrointestinal (GI) tract characterized by an inflammatory process that requires lifelong medication.^274^ Over the past decades, IBD has been regarded as a worldwide public healthcare challenge,^275^ and it can usually be classified into two major forms: Crohn’s disease (CD) and ulcerative colitis (UC). IBD was prevalent in Western countries in the past,^276^ but a significantly increasing incidence has been observed in developing countries more recently.^277^

The pathogenesis of IBD is complicated. Multiple factors constitute a confluence of risk factors for IBD, and they may include genetic and environmental changes that cause dysregulation of the intestinal barrier, immune responses, and microbiota.^278^ It not only affects the GI tract but is also a major causative factor of a number of complications, such as nonalcoholic fatty liver disease (NAFLD) and psoriasis.^279,280^ NAFLD in particular has been considered a leading cause of liver disease in IBD patients,^281^ with a prevalence of 8–40%.^282^ Meta-analyses have shown that patients with psoriasis are 1.70 times more likely to have CD and 1.75 times more likely to have UC.^283^ Therefore, among the numerous complications of IBD, NAFLD, and psoriasis negatively affect the health of patients and are difficult to cure. Thus, they bring a heavy burden to patients and families.

#### Alleviating IBD symptoms: emerging TRP targets

Traditional strategies for the treatment of IBD have relied on the frequent administration of high doses of drugs, including antibiotics, anti-inflammatory agents (such as 5-aminosalicylic acid and corticosteroids), and biological and immunosuppressive agents (azathioprine, 6-mercaptopurine, methionine, cyclosporin-A, and tacrolimus),^284^ to reduce inflammation.^285^ The emergence of monoclonal antibodies as biological therapies has significantly increased the treatment options for IBD.^286^ Numeros TNF-α antibodies, such as adalimumab or certolizumab, are used in the clinical treatment of IBD.^287^ Antibodies targeting IL-12 and IL-23, such as ustekinumab and natalizumab, have also been proposed for clinical approval.^288^ These drugs can effectively relieve early inflammatory symptoms, but their long-term use can cause toxicity and damage organs. These problems may be effectively avoided when certain TRP modulators are used.

#### TRPVs in IBD

Activation of TRPV4 receptors in the GI tract can trigger inflammation.^289^ TRPV4 is expressed and functional in human colon samples, human intestinal epithelial cell lines (Caco-2 and T84), and inflamed colons of mice; 4α-phorbol-12,13-didecanoate (4αPDD) activated TRPV4 in the GI tract and increased the intracellular calcium ion concentration, which ultimately leads to chemokine release and colitis.^290^ Cenac et al.^291^ demonstrated that 4αPDD selectively activated TRPV4 in colonic sensory neurons, which affected visceral nociception and hypersensitivity. Fichna et al.^292^ first observed the significantly higher expression of TRPV4 mRNA in the tissues of IBD patients compared with those of non-IBD patients. Activation of TRPV4 receptors in the GI tract has a pro-inflammatory effect, and selective blockade of TRPV4 in an animal model of IBD alleviated colitis and the pain associated with intestinal inflammation. De Petrocellis et al.^293^ showed that activation and desensitization of TRPV channels mediated intracellular Ca^2+^ concentrations. Interference with TRPV1–4 channel activity and expression may have multiple potential therapeutic applications for IBD.

**Fig. 5:**
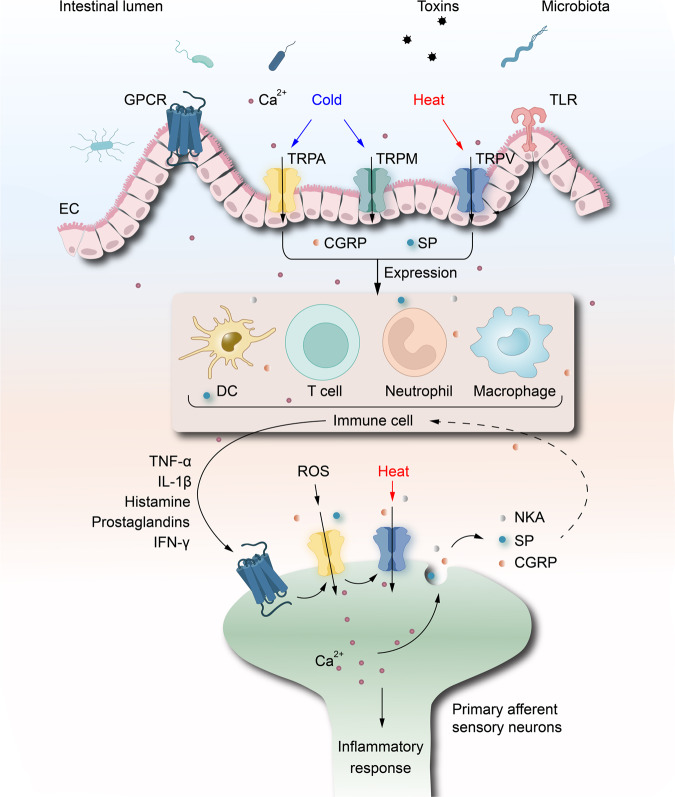
TRP channels in IBD. TRP channels can be stimulated by a variety of factors in the intestinal lumen, including temperature, chemoregulators, and inflammation, and act as auxiliary transducers for GPCR. Interestingly, TLRs play a major role in the regulation of TRPV1 signaling. Stimulation of TRP channels on EC triggers the release of the neuropeptides CGRP and SP, which activate surrounding immune cells and release more inflammatory factors such as TNF-α, IL-1β, histamine, and IFN-γ. These inflammatory factors accumulate at the GPCR of primary afferent sensory neurons. Subsequent activation of TRP channels on neurons triggers the release of neurokinin A, CGRP, and SP, which promote pain perception and inflammatory responses. Created with BioRender.com

Increased TRPV1 nerve fibers in patients with IBD correlated with abdominal pain severity.^294^ Upregulation of TRPV1 protein expression in IBD patients affected nerve endings and immune cells, which suggests the importance of TRPV1 in colonic immune regulation of the inflammatory response and in the pathogenesis of IBD.^295^ Over 20% of patients with IBD have significantly elevated TRPV1 expression in the rectum, which is a key point in mediating inflammatory pain.^296^ The process of producing neuropathic pain in patients with IBD is associated with the activation of TRPV1 because it promotes the release of neuropeptides, such as SP and CGRPs from peripheral terminals (Fig. 5).^297^ Moreover, increased levels of TRPV1 were observed in patients with acute exacerbations of IBD and in experimental models of acute colitis.^298^ Pretreatment of the IBD model with the TRPV1 antagonist JYL1421 reduced visceral hypersensitivity, inhibited colorectal dilation, and improved microscopic colonic inflammation.^299^ All these findings support the idea that TRPVs at least partially participate in the regulation of IBD.

#### Activation of TRPM8 for the treatment of IBD

*TRPM8* mRNA is highly expressed in colonic DRG neurons, and TRPM8 protein is distributed throughout the nerve fibers of the colon wall. Harrington et al.^300^ suggested that TRPM8, which is present in colonic sensory neurons, couples to TRPV1 and TRPA1 and inhibits their downstream chemosensory and mechanosensory effects. In animal models of post-inflammatory colonic allergy, increased TRPM8 expression significantly attenuated hypersensitivity to mechanical stimuli and reduced the symptoms of colonic allergy.^301^

Inflammation in mouse colitis models can be alleviated by the activation of TRPM8. Selective activation of TRPM8 reduces the release of inflammatory neuropeptides, inhibits the release of pro-inflammatory cytokines and chemokines, and prevents the accumulation of leukocytes in the colon.^302^ Inhibitors of phospholipase A2 (PLA2) reduce the response of TRPM8 to cold and agonists, and lysophospholipids as products of PLA2 have the opposite effect on TRPM8.^303^ Notably, PLA2 levels are elevated in patients with CD and UC,^304^ whereas levels of lysophospholipids are clearly decreased.^305^ These changes may also have implications for the function of TRPM8 during colitis.

#### TRPA1 agonists

Ample evidence indicates that TRPA1 plays a major role in the development of IBD.^4^ 2,4,6-Trinitrobenzene sulfonic acid (TNBS) induces colitis with colonic dilation and increases sensitivity to chemical stimuli; TRPA1 expression is upregulated in the colonic afferent DRG neurons.^306^ Intrathecal administration of the TRPA1 antisense oligodeoxynucleotide inhibitor resulted in the downregulation of TRPA1 expression in the DRG and inhibited IBD-induced nociceptive hypersensitivity, colonic dilation, and allyl intracolonic isothiocyanate.^307^ Dinitrobenzene sulfonic acid (DNBS) binds to the intracytoplasmic N-terminal cysteine residue of the TRPA1 protein; thus, effective treatment of DNBS-induced colitis is achieved by antagonizing TRPA1 or genetic deletion of TRPA1, and therefore, TRPA1 is considered a direct target of DNBS-induced IBD.^308^

Application of TRPA1 agonists alleviated DNBS-induced colitis inflammation in mice. Activation of TRPA1 restored the integrity of the colonic epithelial mucosa, inhibited the production of nitric oxide, IL-10, and IFN-γ, and significantly reduced nitrite levels.^309^ These data suggest the significant role of TRPA1 in restoring intestinal mucosal integrity and reducing inflammatory factors after IBD. It may also be used to provide a new therapeutic strategy for the relief of IBD.

#### Complications of IBD: NAFLD

NAFLD is one of the most prevalent complications of IBD,^310^ and it may occur at any stage of the natural course of the disease.^311^ The prevalence of NAFLD ranges from 1.5% to 55% in UC and from 1.5% to 39.5% in CD (with an overall mean prevalence of 23%).^312^ Patients with IBD with NAFLD tend to have stable liver disease that is stable for 4–6 years.^313^ NAFLD is also the most common liver disease, with a prevalence of up to 25% worldwide^314^; thus, it imposes a significant economic burden on patients and leads to a health-related decline in quality of life.^315^ It is characterized by fibrosis, inflammation, fatty deformation, and lipid deposition in hepatic parenchymal cells.

#### TRPV as a therapeutic target in NAFLD

**Fig. 6:**
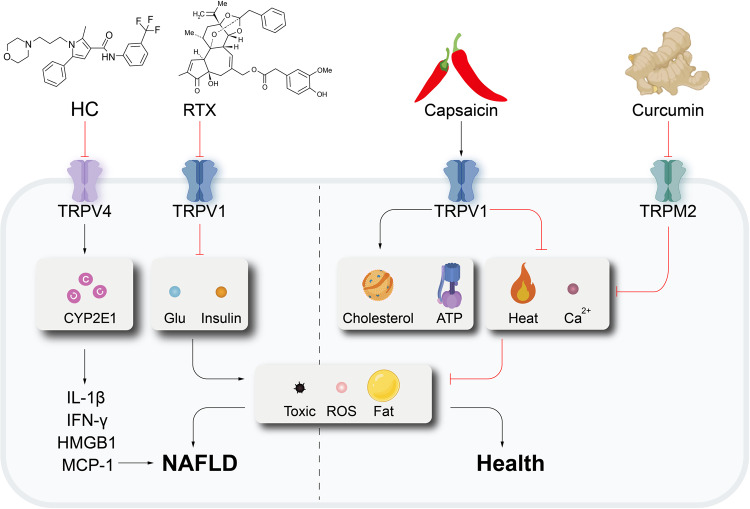
Effect of TRP channels on NAFLD. HC-067047 (HC) inhibits TRPV4 activity, increases CYP2E1 activity, and upregulates IL-1β, IFN-γ, HMGB1, and MCP-1 expressions, which lead to NAFLD. Activation of TRPV1 increases ATP consumption and cholesterol metabolism, reduces thermogenesis and calcium ion concentrations, decreases ROS levels, visceral fat accumulation, and toxicity and ultimately inhibits NAFLD development. Conversely, inhibition of TRPV1 activity is followed by a decrease in serum glucose and insulin, which can exacerbate the disease. Interestingly, curcumin counteracts the development of NAFLD by inhibiting TRPM2, which results in lower calcium ion concentrations and ROS levels and inhibits hepatocyte damage and death. Created with BioRender.com

Activation of TRPV1 increases energy metabolism and prevents the accumulation of visceral fat.^316^ Monoacylglycerols (MGs) with unsaturated long-chain fatty acids are new TRPV1 agonists that along with capsaicin, are found in food and capsaicin to prevent the accumulation of visceral fat. Iwasaki et al.^317^ tested a diet of MGs that activated TRPV1 and found a reduction in the weight of white adipose tissue and lower levels of serum glucose, total cholesterol, and free fatty acids. Activation of TRPV1 also enhanced energy expenditure and increased thermogenesis, which further reduced visceral fat accumulation in mice and prevented NAFLD.^318^ Therefore, activation of TRPV1 is considered an important candidate for reducing the risk of diseases, such as NAFLD (Fig. 6).

Long-term activation of TRPV1 in tissues causes upregulation of hepatic uncoupling protein 2 (UCP2), which effectively prevented high-fat diet-induced NAFLD in mice.^319^ Previous studies have shown that TRPV1 activation by dietary capsaicin resulted in the upregulation of UCP2 expression in a wild-type (WT) mouse liver model but not in *Trpv1* knockout mice. These results suggest that the mechanism by which TRPV1 is activated to prevent exacerbation of fatty liver may be associated with increased UCP2-mediated hepatic β-oxidation.^320^ Thus, dietary capsaicin may serve as a promising intervention for people at high risk of fatty liver disease. However, whether TRPV1 is involved in the regulation of lipid accumulation and the underlying mechanisms involved in this process are currently unclear.

Song et al.^321^ observed that ruthenium red or a synthetic siRNA against TRPV4 inhibited the proliferation of HSC-T6 cells, which caused downregulation of myofibroblast markers, including α-SMA and Col1α1. Seth et al.^322^ reported that TRPV4 responded to the cytotoxic environment of the liver and negatively regulated cytochrome p450 enzymes (CYP2E1). Activation of TRPV4, which releases NO to act on neighboring hepatocytes, inactivates CYP2E1-induced redox toxicity (Fig. 6). TRPV4 acts as a cellular stress sensor in diseased tissues of patients with NAFLD, and thus, it constitutes an endogenous defense molecule that may act as a novel regulator in a protective manner for hepatocytes.

#### TRPM and TRPC channel involvement in NAFLD

Assessment of acetaminophen-induced liver injury based on blood liver enzyme concentrations and liver histology revealed significantly less severe liver injury in *Trpm2* knockout mice than in WT mice.^323^ TRPM2 channels are integral in the mechanism of acetaminophen-induced hepatocellular death. Thus, TRPM2-mediated cell death is an important mechanism in the pathogenesis of NAFLD-induced liver injury (Fig. 6).^324^ However, blockade of TRPM7 inhibits the activation and proliferation of primary hematopoietic stem cells and induces apoptosis in activated cells; ER stress was identified as a possible underlying molecular basis.^325^ TRPM2 and TRPM7 are valuable future therapeutic targets for NAFLD and serve as basis for discoveries related to in this disease.

Arterial flow-regulated diastolic function, which leads to atherosclerosis, is lower in patients with NAFLD than in the healthy population. TRPC3-related endothelial mechanisms that promote NAFLD drive the progression of atherosclerosis. In vitro, TRPC3 is inhibited in hepatic sinusoidal endothelial cells, which makes them less sensitive to ER stress-induced apoptosis; therefore, the pro-apoptotic influence of TRPC3 may increase other fibrotic factors in vivo.^326^ A positive correlation was found among enhanced expression of endothelial TRPC channels, development of early steatohepatitis, and atherosclerotic load in a mouse model of nonalcoholic fatty liver hyperlipidemia fed with a traditional Western-style diet.

Nishiyama et al.^327^ speculated that TRPC3 or TRPC6 may be involved in the fibrotic process of NAFLD. However, systemic deletion of either TRPC3 or TRPC6 does not attenuate liver fibrosis, inflammation, nor steatosis. These findings suggest that TRPC3 and TRPC6 are unlikely to be implicated in liver dysfunction and fibrosis in NAFLD mouse models.^328^ No effective therapy is available for NAFLD, and its exact pathogenesis remains to be elucidated. All these data indicate that TRP channels may be an interesting target for drug development for the treatment of NAFLD.

#### Extraintestinal complications of IBD: psoriasis

A distinct bidirectional association exists between psoriasis and IBD.^329^ At the epidemiological level, patients with psoriasis are at increased risk of developing IBD, and vice versa.^330^ On the skin, TRPs are interesting pharmacological targets because they allow for topical treatment and may reduce the risk of severe harmful effects.^331^ TRP channels are widely expressed in various skin cell types, including keratinocytes,^332^ melanocytes,^333^ nerve endings, and immune cells.^245^ Their functions involve hair growth,^334^ wound healing,^335^ skin cell proliferation and differentiation, immunity,^336^ skin barrier function,^337^ and skin inflammation and pruritus.^338,339^ IBD and psoriasis are relatively common diseases that require long-term treatment. Immunosuppressive drugs are currently the most commonly used treatment in clinical practice and a significant overlap has been observed between effective drugs for the treatment of IBD and psoriasis. The development of potent targeted therapies for TRPs is changing the outlook for chronic inflammatory diseases such as IBD and psoriasis.

#### Remitting effect of inhibition of TRPVs on psoriasis

TRP channels contribute differently to various psoriasis symptoms. Activation of TRPV1 and TRPC4 was found to be detrimental to psoriasis, whereas TRPA1 was found beneficial in preclinical studies.^340^ Lee et al.^341^ reported that the inhibition of TRPV1 activity, which activates ALX/FPR2 receptors, coupled with the control over CGRP release, significantly alleviated psoriasis-like pruritus and inflammation in an imiquimod (IMQ) animal model of psoriasis.

The TRPV1 and NaV1.8 neuroreceptors control the cutaneous immune response by interacting with dermal dendritic cells to inhibit the production of several key effector cytokines in psoriatic dermatitis and regulate the IL-23/IL-17 pathway.^342^ Neurons expressing TRPV1 can mediate itch signaling.^343^ However, intrathecal injection of capsaicin (10 μg) disrupts the central terminals of TRPV1-expressing neurons.^344^ Therefore, we hypothesize that TRPVs are considered novel targets for the treatment of psoriasis by suppressing itching to relieve psoriasis symptoms.

#### Modulation of TRPM and TRPC channels

Cold activation of TRPM8 reduces pruritus caused by psoriasis.^345^ This mechanism possibly occurs through blocking of histaminergic and non-histaminergic pruritic pathways by pharmacological hypothermic cooling.^346^ Activation of TRPM8 also reduces inflammation-induced psoriasis due to its capability to strongly improve edema, inhibit mechanical nociception, and decrease levels of inflammatory cytokines (IL-1β, TNF-α, and IL-6); as a result, the inflammatory response is reduced.^347^ In conclusion, these studies provide strong evidence that TRPM8 is a major cold sensor for amelioration of pruritus and inflammation in various dermatological conditions; it enables the use of TRP channel modulators in the treatment of psoriasis.^348^

In the IMQ-induced psoriasis mouse model, activation of TRPM8 channels stimulated Ca^2+^ responses in cervical DRG neurons, reduced scratching behavior in IMQ mice,^349^ attenuated IMQ-induced morphological changes in lesions, and controlled the infiltration of dermal neutrophils, dendritic cells, and Th17 cells.^350^ Activation of TRPM8 channels also significantly inhibited the generation of iNOS, cyclooxygenase (COX)-2, TNF-α, and IL-6, and prevented the phosphorylation of IκBα, NF-κB p65, ERK, JNK, and p38 MAPKs.^351^ Thus, activation of TRPM8 channels is thought to be effective in ameliorating the symptoms of IMQ-induced pruritus and psoriasis-like inflammation, which make it a promising strategy for the treatment of psoriasis.

The level of 5-HT is higher in the skin of patients with psoriasis than that in healthy individuals.^352^ Specific inhibition of TRPC4 immediately controlled the neuronal activity induced by α-methyl-5HT in dermal nerve preparations.^353^ Interestingly, visual signs of skin inflammation, such as erythema and desquamation, were significantly improved after TRPC4 inhibition and were accompanied by a reduction in epidermal thickness and inflammatory cell infiltration. This effect was achieved by silencing TRPC4 in the DRG and showed an effective reduction in psoriatic pruritus even after repeated applications of IMQ.^354^ Therefore, TRPC4 antagonists may soon be used clinically for the treatment of acute pruritus and psoriasis.

### Respiratory system

Various TRP channels are widely distributed in the lungs and are involved in pathophysiological processes.^355^ A growing body of research suggests that TRP channels may become targets for drug use in the treatment of respiratory diseases.

#### 2019 coronavirus disease (COVID 19)

The COVID-19 pandemic, which is caused by severe acute respiratory disease coronavirus 2 (SARS-CoV-2), has resulted in hundreds of millions of confirmed cases worldwide.^356^ Calcium channel blockers are protective against death from SARS-CoV-2 infection and inhibit the infectivity of SARS-CoV-2 in epithelial lung cells.^357^ Given that coronaviruses require Ca^2+^ ions to enter host cells, Ca^2+^ are considered essential for viral entry into host cells.^357^ Interestingly, most TRP channels are highly permeable to Ca^2+^.^358,359^ Therefore, the researchers hypothesized that TRP channels are involved in certain processes in COVID-19.

The report by Leidinger et al. points to the idea that TRPC6 channels are involved in physiology of normal human lungs and in COVID-19 pneumonia.^360^ They suggested that TRPC6 may exacerbate SARS-2-induced inflammation, but the molecular mechanism remains to be elucidated. Several patients with COVID-19 will develop acute respiratory distress syndrome (ARDS).^361,362^ Khodadadi et al.^363^ found increased expression of TRP cation channels after the treatment of ARDS. Drugs may exert their protective effect against ARDS through TRPs.

Myalgic encephalomyelitis/chronic fatigue syndrome (ME/CFS) has also been defected in some COVID-19 patients.^364–366^ Clinical studies have shown that TRPM3 channel activity is impaired in patients with post-COVID-19 conditions, which suggests impaired ion mobilization; such condition may impede cellular function and lead to chronic post-infection symptoms such as ME/CFS.^367^

Symptoms of COVID-19 associated with TRPA1/TRPV1 include the following: cough,^368^ smell, and taste disturbances,^369–372^ loss of appetite,^373^ nausea,^374^ vomiting,^375^ inflammatory responses, and pain.^40^ However, several clinical studies targeted TRP channels for the treatment of COVID-19. TRPA1 and TRPV1 may provide a breakthrough in the treatment of symptoms of COVID-19.

Given that a common denominator of COVID-19-related symptoms is impaired redox homeostasis, which is responsible for reactive oxygen species (ROS) accumulation, several TRP channels are also involved in oxidative stress. Bousquet et al.^376^ hypothesized the involvement of TRPA1 and TRPV1 in COVID-19. In another direction of thought, harmful stimuli activate TRPV1 and increase the release of SP and pro-inflammatory cytokines,^377^ which exacerbate the symptoms of patients infected with COVID-19.

TRPV4 channels are involved in the recruitment of neutrophils and macrophages during lung injury.^378^ In addition, TRPV4 has been associated with pulmonary edema and pulmonary fibrosis.^379^ Therefore, TRPV4 is a novel target for COVID-19 treatment.^380^ TRPs are involved in pathophysiological processes that largely overlap with the symptoms of COVID-19. Published papers on COVID-19 have proliferated, but the mechanisms underlying the disease and TRPs remain inconclusive. TRP channels represent promising targets for the relief of multiple symptoms of COVID-19.

#### Cough

Cough is a reflex action of the respiratory tract for clearing the upper airways.^381^ It is globally prevalent across all age groups. Stimulation of bronchopulmonary vagal C-fibers by inflammatory mediators can also cause cough.^382,383^ TRPV1 is expressed in the C-fiber sensory nerves of the vagus in the lungs and is involved in the afferent sensory loop of the cough reflex.^384^ Trevisani et al.^385^ noted the pathogenic role of TRPV1 in cough in animal experiments. Iodo-resiniferatoxin (a potent TRPV1 inhibitor) significantly reduced cough induced by inhalation of capsaicin or citric acid in conscious guinea pigs.^386^ Therefore, inhibition of TRPV1 expression is a potential target for cough treatment.

TRPV4 plays a role in increasing sensitivity in the afferent arm the cough reflex.^387^ Selective activation of TRPV4 caused depolarization of airway sensory nerves and cough symptoms in experimental animals. Conversely, TRPV4 antagonists can reverse this phenomenon.^388,389^ In addition, the selective P2X3 antagonist AF-353 inhibits TRPV4-induced neural depolarization and coughing in animals and humans, which suggests that TRPV4 can cause ATP release and relieve cough.^388^ Thus, activation of TRPV4, through the release of ATP is likely to be a pathological cause of the cough.

The TRPA1 receptor (not activated by capsaicin) has become a topic of interest in the field of cough treatment because it is known to be activated by ligands such as acrid smoke.^390^ Birrell et al.^391^ found in normal humans and guinea pigs that inhalation of cinnamaldehyde (a TRPA1 agonist) produced a dose-related increase in cough. Glenmark’s compound GRC 17536, a potent and specific TRPA1 antagonist, effectively suppressed the cough induced by a citric acid challenge in guinea pigs.^392^ A clinical study was subsequently conducted but was terminated due to the lack of clinical efficacy.

TRPM2 may participate in oxidative stress at the cough center by regulating cough.^393^ In the case of TRPM8, menthol prevents coughing by activating TRPM8.^394^ Neurophysiological, immunohistochemical and molecular analyses suggest that the cough suppressant effect of peppermint occurs via a reflex initiated from nasal TRPM8.^395,396^ Based on the properties of TRPs, TRPM8 is undoubtedly one of the targets of cough. Most clinical studies targeting TRPs for the treatment of cough have ended with no drug efficacy. Future research should focus on new strategies to targeting this channel.

#### Chronic obstructive pulmonary disease (COPD) and asthma

Asthma and COPD both cause airway obstruction and are associated with chronic inflammation of the airways;^397^ they are also very common respiratory diseases.^398,399^ A key risk factor for developing respiratory disease is smoking.^400,401^ The use of TRPV1 and TRPV4 antagonists inhibits cigarette smoke-induced ATP release from human bronchial epithelial cells and may be associated with COPD-influenced increases in pulmonary ATP.^402^ Activation of TRPV4 and opening of pannexin 1 channels leads to ATP release, which contributes to the pathogenesis of COPD.^403^ Baxter et al. also noted that TRPV4 gene expression was increased in the lung tissue of COPD patients.^402^ At the genetic level, 7 of the 20 single-nucleotide polymorphisms of TRPV4 tested have been associated with COPD, which suggests a potential relationship between *TRPV4* protein structure and disease pathogenesis.^404^

Contraction of airway smooth muscle in human and guinea pig tissues was observed in vitro with TRPV4 agonists, and this condition may exacerbate asthma.^405^ McAlexander et al. suggested that activation of TRPV4 leads to airway constriction in humans and is entirely dependent on the production of cysteine leukotrienes.^406^ Therefore, activation of TRPV4 may exacerbate symptoms in patients with asthma.

Several endogenous TRPV1 agonists are present in diseased lungs, such as those affected by COPD and asthma. The products of lipoxygenase metabolism of arachidonic acid, such as 12-(S)- and 15-(S)-hydroperoxyeicosatetraenoic acid, are released during asthma attacks and have a stimulatory effect on TRPV1.^407^ Another endogenous agonist of TRPV1, anandamide, is also synthesized in the lungs.^408^ The increased sensitivity of patients with COPD or asthma to inhaled capsaicin may explain the involvement of TRPV1 in the exacerbation of symptoms of these diseases.^409^

In contrast to TRPV1 and TRPV4, little is known about TRPV2.^410^ TRPV2 acts in the early stages of receptor-mediated phagocytosis by macrophages.^411^ Its relevance to alterations in macrophage responsiveness and host defense in COPD still needs to be determined.

Andre et al.^412^ demonstrated that bronchial ring constriction produced by cigarette smoke extracts and acrolein or crotonaldehyde was inhibited by TRPA1 inhibitors. Furthermore, TRPA1 activation and increased Ca^2+^ influx promoted neuropeptide release from the isolated guinea pig airway tissue. These data suggest that airway neurogenic inflammation is mediated via TRPA1 stimulation, and suggest that TRPA1 may play a role in the pathogenesis of COPD.

Notably, *TRPC6* mRNA expression significantly increases in alveolar macrophages from COPD patients, whereas there was no difference has been observed in the expressions of other TRPC subfamily members.^413,414^ TRPC6 is a potential candidate target for COPD, but its role in the regulation of macrophage function under physiological and pathophysiological conditions remains to be determined.

A novel truncated functional TRPM8 variant activated by cold and menthol has been identified in human bronchial epithelial cells.^415^ Whether TRPM8 expression is altered in the airway nerves or airway epithelium of individuals with cold-induced asthma and disease exacerbation remains to be elucidated.^416^

#### Other respiratory diseases

Idiopathic pulmonary fibrosis (IPF) is a prototype of chronic, progressive, and fibrotic lung disease.^417^ Rahaman et al.^418^ determined that TRPV4 activity was upregulated in lung fibroblasts from IPF patients, and mediated the onset of lung fibrosis. TRPV4 also plays an important role in the formation of pulmonary edema and acute lung injury.^378,419^ TRPV4 agonists caused pulmonary endothelial permeability, which was inhibited by the non-selective TRP blocker ruthenium red and was absent in *Trpv4* knockout mice.^420^ Evidence showing that activation of TRPV4 may be involved in disrupting the integrity of the epithelial and endothelial barriers suggests the possible role of TRPV4 in edema formation. Studies also reported that TRPC6 is involved in pulmonary edema formation.^421^ Selective TRPC6 blockers were found to inhibit pulmonary ischemia-reperfusion-induced edema.^422^

A number of TRP channel modulators have entered clinical trials and significant progress has been made in describing the physiopathological function of TRP channels in the respiratory system.

### Nervous system

Neurological disorders are the leading cause of disability and the second leading cause of death worldwide.^423^ TRP channels are widely distributed in neurons and the brain,^424,425^ and are thought to be associated with neurological disorders. They are involved in neurological disorders such as epilepsy, stroke, dementia, anxiety, neurodegenerative diseases, and depression.

#### TRPC

TRPC is involved in chemokine ligand 2-mediated neuroprotection. Specific blockade of TRPC1 and TRPC5 inhibited ERK/cAMP response element binding protein activation and provided neuroprotection.^426^ TRPCs have also been linked to Parkinson’s disease (PD) and Alzheimer’s disease (AD). TRPC1 overexpression may exert PD protection by mediating an inward calcium flow, inhibiting cytochrome c release in mitochondria,^427^ and reducing MPP-induced neurotoxicity and apoptosis.^428^ In AD, each member of the TRPC channel performs a different role. TRPC1, TRPC3, and TRPC4 are found to be involved in neuroprotection,^429,430^ TRPC3 may be involved in the dysregulation of tau proteins in AD, and TRPC5 possibly participates is involved in neurodegeneration.^431^

In addition to these disorders, the TRPC channels are involved in neurological disorders, such as autism, bipolar disorder (BD), and mental retardation.^432^ Griesi-Oliveira et al.^433^ found that the sequence of the *TRPC6* gene on chromosome 11 was disrupted in autistic patients, resulting in haploinsufficient function. Later, they studied the effects of *TRPC6* disruption on pluripotent stem cell-derived cortical neuronal cells. They observed that alterations in neurons of autistic patients were associated with disrupted expression of TRPC6.^432^ A behavioral study of *Trpc6* knockout mice by Griesi et al. found no alterations in social or repetitive behavior, but a reduction in exploratory behavior may be associated with clinical signs of autism.^433^

Evidence from functional studies points to the involvement of TRPC3 and TRPC7 in BD.^434^ Clinical results demonstrated reduced TRPC7 expression in a subgroup of BD patients.^435^ Interestingly, this reduction was inversely correlated with the basal level of Ca^2+^ in B lymphocyte lines (BLCLs) from BD patients. Andreopoulos et al.^436^ treated BD rats with chronic lithium and TRPC3 protein levels were significantly reduced, whereas mRNA levels were not.^437^ In a study by Roedding et al. investigated whether TRPC3 expression and function were affected by oxidative stress in BD and control BLCLs; chronic mitochondrial-generated oxidative stress reduced TRPC3 protein levels and Ca^2+^ influx through these channels, but no significant difference was observed between the two groups.^438^ Thus, TRPC3 is probably not an ideal target for BD.

Depression is a common psychological disorder in the society and is also a neurological disorder.^439^ Hypericin acts as a non-selective neurotransmitter reuptake inhibitor and has antidepressant effects.^440^ It produces a partial antidepressant effect by activating TRPC6 channels, increasing sodium permeability in the presynaptic membrane, reducing the sodium gradient driving neurotransmitter reuptake through the transporter, and increasing the levels of monoamine neurotransmitters in the synaptic cleft.^441^ To a certain extent, depression is also associated with brain-derived neurotrophic factors (BDNF).^442^ TRPC channel-induced calcium transients regulate the density and morphology of dendritic spines in BDNF, to produce antidepressant effects.^441,443^

TRPC channels play an important role in vasospasm in hemorrhagic stroke, neuronal death and survival in ischemic stroke, and thrombin-induced pathological changes in astrocytes, and triggering of stroke by affecting blood pressure and atherosclerosis.^444^ In Ca^2+^ imaging experiments, Ca^2+^ influx through TRPC6 activated MAPK signaling and promoted the neuroprotective chemokine CXCL1,^445^ which protects nerves following stroke. The unique channel characteristics and downstream pathways of TRPCs render them potential new targets for the treatment of neurological diseases.

#### TRPVs

##### *PD.*

To address the accumulation of fibrillar alpha-synuclein in PD patients.^446^ Yuan et al.^447^ found that regulating the opening of TRPV1 channels on the surface of microglia enhanced autophagy, phagocytosis and degradation of α-syn in microglia, which improved the status of PD. TRPV4 contributed to the loss of dopamine neurons and motor deficits in the substantia nigra of PD mice by mediating ER stress and inflammatory pathways.^448^ Furthermore, activation of TRPV1 channels on astrocytes activated endogenous neuroprotective mechanisms in vivo, which prevented active degeneration of dopamine neurons; this condition led to behavioral recovery via CNTF receptor alpha in nigrostriatal dopamine neurons.^449^ These studies provide a new perspective on future molecular targets and gene therapies for the treatment of PD.

##### *AD.*

One of the features of AD mentioned earlier is the deposition of amyloid in the brain.^450–453^ Metabolic facilitation with TRPV1 agonists reduced amyloid lesions and reversed memory impairment in a mouse model of AD.^454^ In addition, the activation of TRPV1 inhibited endocytosis of the α-amino-3-hydroxy-5-methyl-4-isoxazole propionic acid receptor of GluA2 and improved cognition and synaptic function in the AD mouse model.^455^ Furthermore, HC067047 (a TRPV4 inhibitor) enhanced the expression of the neurogenic marker DCX in cognitively impaired mice and increased the levels of the mature neuronal marker NeuN, which indicated the neuroprotective potential for the treatment of dementia with learning and memory loss.^456^

##### *Stroke.*

Activation of TRPV2 induces the release of nerve growth factor (NGF) and regulated blood-brain barrier (BBB) function, which may be associated with ischemic stroke.^457^ TRPV4 alleviates ischemic injury by increasing glutamate and overloading Ca^2+^, thereby reducing neurotoxicity.^458^ Preservation of microcirculation and BBB function shortly after ischemic stroke is a key neuroprotective effect of TRPV4 inhibition, which suggests a contribution of TRPV4 to postischemic brain injury.^459^

#### TRPA1

The toxic effects of amyloid-β on astrocytes triggered by TRPA1 channel activation are key to the progression of AD.^460^ Furthermore, mitochondria have an important role in the pathogenesis of AD.^461^ TRPA1 channels reduces the intracellular Ca^2+^ concentration, apoptosis and intracellular ROS, and this may have a role in the treatment of AD.^462^ TRPA1 activity, if inhibited, will exacerbate astrocytic lesions, inhibit the production of pro-inflammatory cytokines as well as the activity of astrocytes and the transcription factors NF-κB and nuclear factor of calcineurin-activated T cells (NFAT), and play a key role in the pathogenesis of AD.^194^

Prolonged activation of TRPA1 by trans-cinnamaldehyde leads to increased epileptic activity.^463^ In contrast, HC030031 (a TRPA1 antagonist) promotes anticonvulsant effects, and preventes TRPA1 upregulation during seizures.^464^

Increased NADPH oxidase and superoxide dismutase activities and TRPA1 endogenous agonist levels (hydrogen peroxide and 4-hydroxynonenal) were reported in a mouse model of progressive multiple sclerosis-experimental autoimmune encephalomyelitis.^465^ Thus, TRPA1 may be involved in depression and anxiety-like behaviors. TRPA1 also exerts tonic control and promotes anxiety and depression.^466^ Antagonists of TRPA1 are a potential drug category for the treatment of anxiety and mood disorders.

In hypoxia, endothelial TRPA1 channels cause vasodilation, which reduces ischemic damage, a set of phenomena thought to be associated with ischemic stroke.^467^ In combination with these findings, TRPA1 can be included as a candidate for therapeutic targets in neurological diseases.

#### TRPM

TRPM2-mediated cellular and molecular crosstalk with amyloid β peptide or oxidative damage results in the formation of AD lesions.^468^ TRPM2 plays an important role in mediating cell death induced by various oxidative stress-induced pathological factors, including ischemia and reperfusion, the neurotoxic amyloid β-peptide, and MPTP/MPP^+^, which lead to neuronal death in the brain.^324,469^ Activation of TRPM2 alters intracellular ion homeostasis, which leads to abnormal initiation of various cell death pathways, making it an important mechanism in the pathogenesis of ischemic stroke and neurodegenerative diseases.^470^

Hazalin et al.^471^ found that inhibition of TRPM4 expression at the mRNA and protein levels improved cognitive deficits in chronically underperfused rats, but did not affect their motor function. It has also been suggested that TRPM4 plays a key role in the treatment of ischemic stroke.^472^ Chen et al.^473^ examined the link between TRPM4 and ischemic stroke through in vivo assessment and observed that inhibition of TRPM4 improved BBB integrity after reperfusion in ischemic stroke.

The TRP channel may represent a promising therapeutic strategy for neurological disorders. Future research and development should focus on the TRPC channel because it is more widely distributed in the nervous system and existing studies are not sufficiently advanced.

### Cardiovascular system

Given the distribution and function of TRP channels in excitatory and non-excitatory cells of the cardiovascular system, they can be a potential therapeutic target for CVD.^474^ Different TRP family members have various potential functions in CVD, and are mainly involved in hypertrophy, heart failure, arrhythmias, and vascular regulation.^16^

#### Cardiac hypertrophy and heart failure

Cardiac hypertrophy and heart failure are closely related. Ang II-induced cardiac hypertrophy is the key to the pathological process of heart failure.^475,476^ Using siRNA, Onohara et al.^477^ demonstrated that TRPC3 and TRPC6 were required for Ang II-induced NFAT translocation in cultured rat myocytes, which is a key step in cardiac hypertrophy.^478^ Furthermore, Ang II stimulation resulted in a significant increase in apoptosis in cultured neonatal rat cardiomyocytes transfected with *TRPC7*, accompanied by a decrease in atrial natriuretic factor expression and destruction of actin fibers.^479^ Ang II/phenylephrine-infused *TRPC3* transgenic mice showed increased activation of NFAT in vivo, cardiomyopathy, and increased hypertrophy following stimulation with neuroendocrine agonists or pressure overload.^480^ Mice lacking TRPC1 channels maintained cardiac function, which prevented cardiac hypertrophy, when subjected to hemodynamic stress and neurohormonal overload.^481^ TRPV3 expression also increased in Ang II-induced cardiomyocyte hypertrophy in vitro, accompanied by an increased intracellular calcium ion concentration, which promoted the expression of calcineurin and phosphorylated CaMKII protein and enhanced nuclear translocation of NFATc3.^482^

Feng et al.^483^ observed clinically that TRPM4 currents were more than twofold larger in fibroblasts from heart failure patients than from controls, and this finding implies the possible role of TRPM4 in cardiac fibrosis in heart failure. Moreover, dietary activation of TRPV1 by capsaicin may prevent the cardiac hypertrophy caused by a high-salt diet by improving cardiac mitochondrial dysfunction.^484^

#### High blood pressure

High blood pressure is associated with vasoconstriction.^485,486^ Evidence showing the involvement of TRP channels in the regulation of vasoconstriction is lacking. Furthermore, impaired TRPV1 signaling may contribute to the pathogenesis of hypertension.^487^ Anandamide activates TRPV1 on perivascular sensory nerves, which leads to the release of CGRP to reduce vasoconstriction.^488^ Anandamide also inhibits vasoconstriction by activating the TRPV1 receptor in the endothelium, which causes the acute release of NO.^489^

TRPV4 and TRPM4 also have a regulatory effects on systemic blood pressure. Plasma adrenaline concentrations and urinary excretion of catecholamine metabolites, which may reduce hypertension, greatly increased in *Trpm4* knockout mice.^490^ Furthermore, in *Trpv4* knockout mice, the odds of hypertension induced by inhibition of NOS were greater, which was mainly related to the involvement of TRPV4 in vasodilation,^491^ this finding suggests the vasoprotective effect of endothelial TRPV4.

#### Atherosclerosis

Atherosclerosis is the process of plaque formation that leads to the development of a number of CVDs.^492,493^ In addition, atherosclerosis may be associated with the apoptosis of macrophages.^494^ Tano et al. discovered that *Trpc3* knockout mice exhibited reduced macrophage apoptosis and necrosis and increased collagen content.^495^ Specific inhibition of TRPA1 exacerbated oxidized low-density lipoprotein-induced lipid accumulation in macrophages, and atherosclerotic lesions and hyperlipidemia aggravated.^496^ Upregulation of TRPM2 enhanced 5-HT vascular reactivity during the development of atherosclerosis.^497^

#### Vascular complications

Autosomal dominant PKD is caused by mutations in *PKD1* and *PKD2* and often associated with vascular abnormalities.^498,499^ *PKD1* and *PKD2* encode polycystins TRPP1 and TRPP2, respectively.^119^ Abnormal intracellular Ca^2+^ regulation associated with *PKD2* haploinsufficiency reduces vascular smooth muscle cell contractions.^500^ This condition is essential for maintaining the structural and functional integrity of blood vessels.^501^ Overall, these observations suggest that TRP channels may be an avenue for mitigating CVD.

### Endocrine system and metabolism

TRP channels also have a wide range of physiological functions in the endocrine system and metabolism, including in diseases such as cancer, obesity, kidney disease, metabolic syndrome, and diabetes.^181,502,503^

#### Cancer

TRP channels play a key role in cancer progression.^504^ Changes in the expression of TRP channels have been found in a number of cancers, including prostate,^505^ breast,^506^ colorectal,^507^ pancreatic,^508^ liver,^509^ and gastric cancers.^510^ Such finding was mainly due to the inhibition of cell migration and invasion,^511^ and tumor cell growth and proliferation caused by TRP channels.^512^ Researchers need to further elucidate the functional role of TRP channels in cancer and their changes.

#### Obesity

Obesity is characterized by excessive fat accumulation in the body.^181^ Notably, TRP channels are expressed in adipose tissue.^513^ In response to exogenous stimulation, TRP channel receptors activate the sympathetic nervous system norepinephrine brown adipose tissue axis to enhance thermogenesis and thereby suppress obesity.^514^ Oral capsaicin reduces abdominal fat^515^ and enhances brown fat thermogenesis, white fat browning,^516^ fatty acid β-oxidation,^517^ and energy expenditure in obese patients.^518^ The function of these TRP channels identifies them as potential targets for obesity.

#### Kidney disease

TRPC6 is expressed in glomerular podocytes. Increased expression levels of TRPC6 were observed in patients with focal segmental glomerulosclerosis (FSGS),^519^ membranous nephropathies,^520^ minimal change nephrosis,^521^ and diabetic nephropathy.^522^ TRPC6 mutations are involved in FSGS at the genetic level.^523^ TRPC 6 plays a cytoprotective role in membranous nephropathy.^524^ Aberrant expression of TRPC6 channels may be an important causative factor in kidney disease, but its clinical application needs further study.

#### Metabolic syndrome

Metabolic syndrome is a common metabolic disorder that results from the increased prevalence of obesity.^525^ As mentioned earlier, TRP channels are involved in CVD and obesity. The dysfunction of TRP channels is likely to contribute to increased cardiovascular risk in patients with metabolic syndrome.^526^ Although no experimental evidence indicates that TRP channels directly affect metabolic syndrome, TRP channels can be investigated as a medicinal target because of their role in related disorders.

#### Diabetes

TRP channels are related to several aspects of the pathology of diabetes, including insulin release from pancreatic beta cells and secondary diseases such as diabetic neuropathy, nephropathy, and vasculopathy.^527^ Chung et al.^528^ observed that the gene expression of *TRPC4* was upregulated by 22%, and the protein expressions of TRPC1 and TRPC6 were downregulated by 50% in blood vessels of diabetic patients compared with non-diabetic controls. They suggested that diabetes affected capacitive calcium entry by regulating TRPCs.^528^ In addition, activation of TRPV4 affects insulin secretion and influences the level of insulin mRNA in cells.^529^ TRP channels have distinct clinical potential for the treatment or prevention of diabetes and its complications. However, given that TRP channels have a number of conflicting indications, clinical drug development will face a number of challenges.

### Urogenital system

Preclinical studies suggested that modulators of TRPs improve genitourinary disorders,^530,531^ including neurogenic bladder,^532^ interstitial cystitis,^533^ and overactive bladder. Extracellular ATP-gated P2X receptors are trimeric non-selective cation channels that are important for nerve conduction.^534^ After posterior tibial nerve stimulation of neurogenic bladder in rats, inflammatory cell infiltration in bladder tissue was significantly reduced, and TRP and P2X expressions in the bladder and DRG were partially restored.^535^ Clinical studies have revealed significantly increased levels of *TRPM8* mRNA and protein expression in bladder tissue from patients with interstitial cystitis/bladder pain syndrome.^536^ In addition, the secretion of inflammatory mediators and oxidative stress by TRPA1 into the periphery may influence the pathogenesis of interstitial cystitis/bladder pain syndrome.^537^

TRPV1 plays a role in the pathophysiology and treatment of overactivity of the detrusor muscle.^538^ TRPV1 expression was significantly elevated in the urothelium of patients with overactive bladder disease.^539^ NGF-induced bladder overactivity depends on the interaction of NGF with TRPV1.^540^ In addition, selective antagonism of TRPM8 inhibits the exaggerated activity of mechanosensitive bladder C-fibers and reduces bladder overactivity.^541^ These studies suggest that TRP channel modulators may be of value in urogenital disorders.

### Immune regulation

Growing evidence demonstrates functional TRP channel expression in immune cells with important implications for immunology. TRP channel activation induces immunomodulatory effects on multiple levels.^542^

IL-31 is a key neuroimmune link between TH2 cells and sensory nerves in the generation of T cell-mediated pruritus.^543^ IL-31-induced pruritus was significantly reduced in *Trpv1* and *Trpa1* knockout mice.^544^ This finding suggests that TRPV1 and TRPA1 are involved in immune response.

To date, the expression of TRPA1 in immune cells has been poorly studied. Expression of TRPA1 was upregulated in atherosclerotic macrophages from mice deficient in lipoprotein E (*Apoe*^*−/−*^).^496^ Selective inhibition of TRPA1 increased the levels of serum triglycerides, high-density lipoprotein, total cholesterol, pro-inflammatory cytokines, and macrophage inflammatory protein-2 (MIP-2) in *Apoe*^*−/−*^ mice.^496^ This result suggests that TRPA1 performs a regulatory function in macrophage foam cell cholesterol metabolism. Activation of TRPA1 also stimulated the release of TNF-α and IL-8 from the human macrophage line U937.^545^

In RAW 264.7 macrophages, the TRPM8 agonist icilin activated TRPM8-like channels.^546^ In vitro, anti-inflammatory effects were produced in mouse peritoneal macrophages by activation of TRPM8.^547^ Activation of TRPM8 also inhibited the production of pro-inflammatory cytokines in human monocytes and lymphocytes in vitro.^548^

TRPV1 is activated in bone marrow-derived macrophages, which attenuates lipid accumulation and reduces the production of monocyte chemoattractant protein (MCP-1), MIP-2 and IL-6.^549^ Activation of TRPV1 in RAW264.7 cells in an inflammatory state inhibited the release of iNOS and NO, COX-2 and PGE2.^550^ Acharya et al.^551^ observed that activation of TRPV1, which induces anandamide production by myeloid cells acting on CB2 receptors, increased the number and immunosuppressive function of regulatory CX3CR1hi macrophages.

TRPV4 is expressed and functions in immune cells, including macrophages, monocytes, neutrophils and T cells.^552^ In alveolar macrophages of WT mice, activation of TRPV4 caused calcium influx and superoxide and nitric oxide production, but this phenomenon was absent in *Trpv4* knockout mice.^419^ Majhi et al.^553^ revealed that in vitro activation of TRPV4 in T cells led to T cell proliferation and production of pro-inflammatory cytokines IFN-γ, TNF-α and IL-2. These results suggest that TRP channels are involved in the coordination of immune response in immune cells.

TRP channels are also involved in the pathogenesis of immune-mediated chronic inflammatory diseases.^554^ They regulate macrophage polarization,^555^ chemokine and cytokine production,^556^ phagocytosis,^411^ and proliferation and survival.^557^ In the future, the expressions of TRP channels in immune cells and the signaling pathways for TRP channel activation in different immune-mediated diseases must be clarified to identify new molecular mechanisms for the treatment of these diseases.

## Therapeutic interventions

TRP channels have important functions in diseases. Thus, the use of TRPs as a drug target has therapeutic potential.

### Pharmaceutical interventions

In recent years, several modulators of TRPs of chemical, biological, and natural origin have entered clinical trials and they have been used to treat inflammatory, neuropathic, and visceral pain although the therapeutic mechanism of action of these compounds is unclear.^153^

#### TRPV1 agonists and antagonists

Various TRPV1 antagonists have been evaluated at various stages of clinical trials for different indications. TRPV1 antagonists show promise for pain treatment. ABT-102, a structurally novel TRPV1 antagonist, has been tested in phase I clinical trials for pain associated with inflammation, tissue damage, and ischemia.^558^ ABT-102 was clinically well tolerated by repeated dosage but caused a short-term increase in the mean core body temperature in healthy volunteers.^559^ Phase II clinical trials have been completed for the use of AZD1386, also an antagonist of TRPV1, for neuropathic pain, dental pain, nonerosive reflux disease, and osteoarthritis.^560^ However, in patients with osteoarthritis and nonerosive reflux disease, the clinical trial was terminated because there was no significant reduction in pain was observed.^561,562^

Capsaicin in chili peppers bestows the sensation of spiciness and is a common component found in the fruits of *Capsicum*.^157,563^ It is a classical TRPV1 agonist. Studies have reported the beneficial effects of capsaicin acting on TRPV1 in obesity, cardiovascular and GI, various cancers, neurogenic bladder, and skin diseases.^564^

A randomized trial assessing the effects of TRPV1 antagonist SB705498 on pruritus induced by histamine and cowhage in healthy volunteers has progressed to phase I.^565^ However, the results of SB705498 on atopic dermatitis showed that it is not clinically relevant for pruritus due to dermatitis. SB705498 has also been investigated for the treatment of migraine (ClinicalTrials.gov: NCT00269022), toothache (ClinicalTrials.gov: NCT00281684) and rectal pain (ClinicalTrials.gov: NCT00461682), all of which have now completed Phase II clinical trials.^566^ The results of these clinical studies have not been published to date.

Phase II clinical trials of XEND0501 for the treatment of chronic cough and COPD (ClinicalTrials.gov: NCT022233699 and NCT022233686) have been completed. Although XEND0501 was well tolerated, it was not clinically meaningful for patients with refractory cough.^567^

PAC-14028 completed a Phase II clinical trial for the topical treatment of pruritus, rosacea, and atopic dermatitis (ClinicalTrials.gov: NCT02052531, NCT02052999 and NCT02583022, respectively).^568^ In 2019, the results of the clinical Phase IIb trial of PAC-14028 cream met safety and efficacy metrics.^569^ In addition to the TRPV1 antagonists mentioned above, numerous chemical, biological and natural agents, including NEO-6860, JNJ-39439335 and others, have been introduced in clinical trials.

#### TRPA1 antagonists

Given its involvement in pain, itching, and respiratory syndromes, TRPA1 has been pursued as a promising drug target.^570^ GDC-0334 is a chemical-derived drug that reduced TRPA1 agonist-induced skin blood flow, pain and pruritus in a phase I clinical trial (ClinicalTrials.gov: NCT03381144).^571^ GDC-0334 provides a therapeutic basis for assessing TRPA1 inhibition as a clinical treatment for asthma.

In 2012, Glenmark selected GRC 17536 for clinical development as a TRPA1 antagonist. In September 2014, Glenmark announced positive results obtained in the Phase II clinical trial.^392^ GRC 17536 had statistically significant and clinically relevant response in patients with moderate to severe painful diabetic neuropathy.^572^ GRC 17536 remains the only TRPA1 antagonist to have completed a Phase IIa study to date.^573^

In 2015, Orion announced that the TRPA1 antagonist ODM108 (acetylene-containing compound) entered phase I clinical trials for neuropathic pain.^570^ However, complex non-linear pharmacokinetics emerged and the clinical trial was discontinued in 2016. A key point of progress in TRPA1 research is the efficient oral utilization of the substance. A problem with all TRPA1 antagonists developed to date is that they are highly lipophilic and relatively insoluble. Addressing this issue is necessary for the drug development involving TRPA1.

#### Regulation of TRPM8

The TRPM8 channel is responsible for the sensation of cold environmental temperatures, it is also clinically associated with pain, itching, and obesity. PF-05105679 is a moderately potent TRPM8 blocker that has been evaluated for the treatment of cold pain sensitivity.^574^ Winchester et al.^575^ observed in clinical trials of PF-05105679 that the compound showed significant pain inhibition in the cold press test and no effect on core body temperature was observed. These results suggest that TRPM8 plays a role in acute cold pain signaling, and its dose does not result in a lower than normal body temperature.

Menthol is a natural source of TRPM8 agonists. Topical menthol activates nociceptors on the skin and then desensitizes them.^576^ When topically applied, menthol can also act as a vasorelaxant, but the mechanism is unclear.^577^ Menthol is also used clinically for migraines, neuropathy, and muscle pain. It provides a cooling effect, leaving human subjects with a cool sensation on the skin and significantly less discomfort than ice.^578^

Another TRPM8-related compound, isopulegol (Phase II, ClinicalTrials.gov: NCT02193438), had no positive biomechanical or neurophysiological effects on patients with oral dysphagia due to stroke or neurodegenerative disease no significant adverse events were observed.^579^

Peppermint oil is another TRPM8 agonist of a natural origin. Winchester et al.^575^ investigated the role of peppermint oil in a mouse model of colitis. The results showed that peppermint oil, activated TRPM8 and the treated mice showed improvements in macroscopic and microscopic parameters and reduced weight loss during colitis.^575^ In addition, Peiris et al. observed that the use of TRPM8 agonist icilin for irritable bowel syndrome resulted in reduced release of inflammatory cytokines IL-1β, IL-6, and TNF-α in patient biopsies.^580^ These results suggest that specific activation of TRPM8 may alleviate experimental colitis and irritable bowel syndrome and may be a promising additional therapy for the treatment of IBD.

#### Other medicinal TRP uses

TRPV1, TRPA1, and TRPM8 are currently being studied in clinical trials, but clinical studies of other channels should not be overlooked. A TRPV4 inhibitor, GSK2798745 was launched by GlaxoSmithKline as a clinical candidate.^581^ In 2019, GSK2798745 underwent its first clinical study (ClinicalTrials.gov: NCT02119260). Positive results were obtained; GSK2798745 was well tolerated in healthy volunteers and in patients with stable heart failure, which allowed further evaluation in long-term clinical studies of heart failure and in other indications.^582^

Preclinical studies have provided a certain rationale to support the development of TRPV3 antagonists for the treatment of inflammatory skin diseases, pruritus and pain.^583^ However, to date, only one TRPV3 inhibitor (GRC15300) has entered clinical trial targeting osteoarthritis and neuropathic pain.^584^ Moreover, by the end of 2013, these trials were discontinued. The Sanofi–Glimark agreement was terminated in 2014, and no further developments have been reported since then.^584^ Although numerous TRP channel drugs have entered clinical trials, only a few have been approved by the US Food and Drug Administration (FDA) (Table 4). In summary, researchers have made progress in understanding the role of TRPs in health and diseases. TRPs show promise as therapeutic targets for the treatment of inflammation, pain, itching, and other conditions.

**Table 4:** FDA-approved TRP channel-targeting drugs

Drug	Function	Target	Drug source	Adverse events	Ref.
Menthol	Antipruritic, analgesic	TRPM8, TRPA1	Natural/chemical synthesis	Increased tobacco smoke toxicity; induced acute respiratory irritation	^160^
Resiniferatoxin	Control of pain associated with bone cancer in dogs	TRPV1	Chemical synthesis	Lethargy, loss of appetite	^688^
NGX-4010	Relief of neuralgia after shingles	TRPV1	Dermal patch containing 8% capsaicin	/	^689^
Eugenol	Sedation	TRPV3, TRPA1	Natural/chemical synthesis	/	^690^

### Physical interference methods

Numerous methods of physical interference are used for TRP channels, and they include moxibustion, acupuncture, photothermal therapy, and electroacupuncture. Moxibustion, acupuncture, and electro-acupuncture are all traditional Chinese medicine treatments.^585^ These methods are applied to the body surface to avoid the adverse events associated with medication.^586^ They are mainly used clinically for the relief of pain and inflammation.^586,587^

The effectiveness of moxibustion therapy is reportedly determined by the activation of TRPV1–4.^588^ In one study, moxibustion treatment of *Trpv1* knockout mice did not produce the significant increase in thermal pain thresholds exhibited by normal mice.^589^ This result suggested that TRPV1 was involved in the process of increasing the thermal pain threshold in the moxibustion-stimulated mouse spinal cord at L5 and L6.^589^ In visceral nociceptive rats, the expression of TRPV1 and heat shock protein 70 was increased. This increase was reversed by moxibustion treatment, which also significantly reduced abdominal withdrawal reflex scores and increased the pain threshold pressure.^590^ Pain treatment of moxibustion may be accomplished by downregulating TRPV1 expression.

Acupuncture significantly increases TRPV1 expression in subcutaneous nerve fibers and modulates TRPV channels in the brain and spinal cord.^591^ Acupuncture also activates the expression of TRPV1 and TRPV4 in the anatomical layers of the Zusanli acupoint (ST36), including the muscle and epithelial layers.^592^ Given that the components of calcium wave propagation, which occurs during acupuncture are also expressed in the TRPV1-rich ST36 anatomical surface, TRPV1 may be involved in the analgesic effects associated with acupuncture.^592^ The researchers used knockout mice to further validate the relationship between TRPV channels and electroacupuncture treatment. Huang et al.^593^ noticed that in *Trpv2* knockout male mice, acupuncture-induced analgesia was suppressed, and activation of mast cells in stimulated acupoints was reduced.

Analgesic effects similar to those observed in acupuncture treatment were also detected in electroacupuncture treatment and mainly related to TRPV channel modulation.^594–596^ Electroacupuncture may improve acid–base-induced chronic pain through its action on TRPV1 and related molecular pathways.^597^ In addition, inhibition of TRPV1 expression by electroacupuncture plays a role in the neuroinflammatory regulation of PD, and downregulation of cytokine levels.^598^ For the pain treatment of cancer patients, electroacupuncture treatment downregulated the overexpression of TLR4, myeloid differentiation primary response 88, and TRPV1 in the DRG.^599^ This condition reduced the activation of spinal glial cells and thus relieved pain. Electroacupuncture modulation of TRPV1 for CVD has also been reported. Electroacupuncture pretreatment enhanced TRPV1 and CGRP signaling, downregulated NF-κB p65 protein expression, reduced the myocardial inflammatory response status, improved acute myocardial ischemic injury and reduced myocardial infarct size.^600^

With the development of scientific research technology, nanoparticles are becoming more widely used. Gold nanorods are pseudo one-dimensional rod-shaped nanoparticles that can be used for thermal therapy when exposed to near-infrared radiation.^601^ Zhou et al.^602^ showed that gold nanorods activated TRPV1 channels through photothermal conversion during exposure to near-infrared irradiation. This nano-loaded anesthetic can produced selective, long-lasting regional anesthetic effects in a rat model of sciatic nerve block. In addition, semi-conducting photothermal nano loads of capsaicin were followed by a photothermal trigger to induce release. The results showed high local concentrations of TRPV1 agonist at the tumor site.^603^

Current physical interventions in TRP channels focus on electroacupuncture and acupuncture for the treatment of diseases. Based on several properties of the TRP channels, we believe that influencing the TRP channels through cold and heat compresses to relieve pain and inflammation is a promising direction to be confirmed by researchers.

### Therapeutic application limitations

The clinical development of TRP channel modulators has provided a scientific basis and new insights into the further use of TRP channels as biomarkers for disease treatment. Despite the appeal of TRP channels in the clinical treatment of pain and inflammation, a number of challenges remain.

In clinical studies, heat and pain are the most common adverse effects of treatment targeting TRP channels. In a study of the effect of inhibition of TRPM8 channels on human pain, the effect on core body temperature and experimental cold pain was assessed by the cold pressor test after PF-05105679 administration.^575^ A total of 23% and 36% of volunteers (at doses of 600 and 900 mg, respectively) reported hot sensations mainly in the perioral region; two volunteers showed intolerance towards the treatment.^575^

Capsaicin has long been considered clinically effective for the treatment of bladder disorders; however, capsaicin in the bladder is unacceptable for a number of patients because it causes burning pain.^604^ Capsaicin also causes profuse sweating and a burning sensation on the mucous membranes of the tongue and mouth, which dissipates after repeated challenges (“desensitization”).^605^ This desensitization is also known as a persistent refractory state following a painful reaction,^532^ which can last for up to several months.^606^

Most antagonists of the TRPV1 channel elevate body temperature. Most patients had a slight increase in body temperature after oral administration of AZD1386 (mean value of 0.4 °C), and only one exceeded 38 °C.^607^ In addition, Amgen’s Phase 1b clinical trial using AMG 517 in dental pain elicited a prolonged (1–4 days) and significant febrile response (up to 40.2 °C) in human volunteers.^608^

TRPV1 channel antagonists can elevate the threshold of toxic heat sensation and lead to burns. Previously, Merck/Neurogenics developed MK-2295 for the treatment of pain and cough.^609^ This compound significantly increased the threshold for toxic heat pain in humans, and several volunteer subjects even perceived scalding water as cold.^610^ Similar results were observed by AstraZeneca and GlaxoSmithKline on a TRPV1 channel antagonist (SB-705498).^607^ In addition, mild burns on the mouth (gums) and skin were noted in patients using the TRPV1 channel antagonist JNJ-39439335.^611^

As TRP channels are expressed in various tissues, systemic administration in the clinical setting may cause varied adverse effects. Among these effects, dizziness, hypertension, heat tremors, headache, nausea, chills, decreased body temperature and paresthesia are more common and should be the focus of researchers (Table 5). Volunteers treated with ticlopidine, clopidogrel and prasugrel (TRPA1 agonists) often complained of GI discomfort such as nausea, vomiting, and diarrhea.^612^ Activation of TRPV1 by RTX has antiemetic and hypothermic effects, but it can also cause hypertension.^613^ The most frequently reported events in subjects taking NEO6860 (TRPV1 antagonist) orally were feeling of hotness, headache, paresthesia, nausea and dizziness.^614^ Therefore, current clinical research can focus on the topical delivery of TRP channel modulators, including topical patches, mucosal sprays, and nano-delivery systems.

**Table 5:** Clinical trials related to TRP channel-targeted therapies

Target	Drug	Indication	Function	Clinical progress	Adverse events/reasons for terminating	Developer	Organization / NCT number	Ref.
TRPV1	Resiniferatoxin (RTX)	Severe osteoarthritic pain, chronic intractable pain, cervical cancer	Reduce pain and improve mobility	Phase II	Destroys nociceptive nerve endings and produces reversible analgesia	Sorrento Therapeutics	National Institute of Neurological Disorders and Stroke	^691^
	AZD1386	Osteoarthritis	/	Phase II	No significant pain decrease; some patients had increased liver enzymes (ALT and AST) after receiving treatment	AstraZeneca	NCT00878501 NCT00976534	^562^
		Chronic nociceptive pain, pain after molar extraction	Rapid, short-acting analgesia	Phase I	Dizziness, headache, nausea, chills, fever	Lifetree Clinical Research, Salt Lake City	Compass Institutional Review Board, Mesa, AZ, USA NCT00736658	^570^
	JNJ-39439335	Knee osteoarthritis	Analgesic	Phase II	Thermal hypoesthesia, paresthesia, feeling cold, minor thermal burns	Mavatrep	NCT01006340 NCT00933582 NCT01343303	^692^
	NEO6860		Analgesic	Phase II	Feel hot, headache, nausea, dizziness, fatigue, hypoesthesia, and increased blood pressure	NEOMED Institute	Central ethics committee (IRB Services, Aurora, Ontario, Canada) NCT02712957 NCT02337543	^693^
	SB-705498	Refractory chronic cough, allergic rhinitis, non-allergic rhinitis	Cough suppression	Phase II	No effect on spontaneous coughing frequency	GlaxoSmithKline	The research ethics committee (11/H1008/2); NCT01476098 NCT01424514 NCT01424397	^694^
	XEN-D0501	Refractory chronic cough, type 2 diabetes mellitus	Inhibited capsaicin-induced cough	Phase II	Dysgeusia, thermal hypoesthesia, hyperhidrosis, oral paresthesia, headache	University Hospital of South Manchester; and Belfast City Hospital	Research ethics committee (REC North West-Liverpool East reference 14/NW/0211) NCT02233699 NCT02233686	^567,695^
	Capsaicin	Neurogenic bladder	Reduced urinary frequency and improved overactive or hypersensitive bladder	Phase II	Its utility is still uncertain	/	/	^564^
		Peripheral neuropathic pain	Significant improvement in pain attacks, sleep duration, and sleep quality	Phase III	Erythema, pain, application site swelling, nausea, pruritus, hyperesthesia	/	The Ethics Committee of the Friedrich–Alexander University, Erlangen–Nuremberg (Re.-No.4458)	^696^
	PAC-14028	Atopic dermatitis, rosacea	Improved atopic dermatitis symptoms and decreased sleep quality	Phase III	Infections, infestations, nasopharyngitis, rhinorrhea, pain, cough, pyrexia, wound secretion, nausea, vomiting	AmorePacific Corporation R&D Center	NCT02757729 NCT02965118 NCT02052531 NCT02052999 NCT02583022	^569,697^
	AMG517	Pain associated with inflammatory diseases and cancer	Plasma concentration-dependent hyperthermia	Phase Ib	Febrile reactions	Amgen	/	^698^
	ABT-102	Pain	Analgesic	Phase I	Feeling hot or cold, hot flushes, altered taste sensation, oral hypoesthesia, dysesthesias	Abbott Laboratories	NCT00854659	^699,700^
	SYL1001	Dry eye disease	Reduce eye pain and conjunctival congestion	Phase II	The effect is not obvious	Sylentis, Madrid, Spain	NCT01438281 NCT01776658 NCT02455999	^701^
	BCTC	Tissue acidosis due to ischemia	Reduce acid-induced pain	Phase II	/	Medical University of Vienna	Ethics committee of the Medical University of Vienna	^702^
	/	IBD, IBS	Increased TRPV1 nerve fibers are seen in quiescent IBD with IBS-like symptoms together with a correlation to pain severity	/	TRPV1 contribute to the pathophysiology of ongoing pain and visceral hypersensitivity of patients	/	/	^294^
TRPV4	AF-219	Refractory chronic cough	The frequency of the patient’s cough was reduced by 75%	Phase II	Taste disturbances, nausea, oropharyngeal pain, headache, salivary hypersecretion, cough	The North West Lung Centre at the University Hospital of South Manchester	NCT01432730	^703^
	GSK2798745	COVID-19, congestive heart failure, chronic cough	Well-tolerated	Phase I	Headache, nasopharyngitis, dizziness, fatigue, nasal congestion, dyspepsia, and feeling abnormal	GlaxoSmithKline	NCT02119260	^581,582,704^
TRPV3	Eugenol	Hair growth disorders, oral irritation	Self-desensitization of oral irritation, enhancement of innocuous warmth, enhancement of heat pain	/	Heat pain; reduced detection of weak tactile stimulation	University of California	The University of California Davis Human Subjects Internal Review Board	^608,705^
	GRC15300	Osteoarthritis, neuropathic pain	Since 2014, no further development has been reported	Phase II	/	Sanofi-Glenmark	/	^249,584^
	Carvacrol	Postburn pruritus	Regulation of cell migration	Phase II	Itch	Hangang Sacred Heart Hospital, Hallym University, Seoul, Korea	/	^706^
	/	Olmsted syndrome, erythromelalgia	TRPV3 mutations lead to Olmsted syndrome	/	/	Université Paris Descartes	/	^707^
TRPV2	Tranilast	Heart failure with reduced ejection fraction	Reduced TRPV2 expression and decreased brain natriuretic peptide levels	/	Decreased renal function, increased heart rate, and increased premature ventricular contractions	National Hospital Organization Toneyama National Hospital, Japan	/	^708^
	Probenecid	Heart failure	Improves cardiac function and increases force development and calcium sensitivity at the cardiomyocyte level.	Phase I	Gastrointestinal discomfort	University of Cincinnati, Cincinnati, OH	NCT01814319	^709^
TRPA1	GRC17536	Painful diabetic polyneuropathy	Significant reduction in pain scores	Phase II	Bioavailability/pharmacokinetic aspects	Glenmark	/	^572^
	GDC-0334	Asthma	Reduce dermal blood flow, pain, itch	Phase I	Mild headache	AbMole	NCT03381144	^571,710^
	Methylglyoxal	Diabetes, multiple sclerosis	Prevention of cognitive decline	Phase I	Hyperalgesia, pain	/	The Medical Ethical Committee of the Jessa Hospital and of Hasselt University, Hasselt, Belgium	^711,712^
	ODM-108	In healthy volunteers revealed no central nervous system toxicity issues	/	Phase I	/	Orion Corporation	NCT02432664	^570^
	Cinnamaldehyde	Oropharyngeal dysphagia, cough	Improved swallowing response, induced cough	Phase II	Gastrointestinal disorders (diarrhea and vomiting), cough, mild nausea	Nestlé Research	NCT02193438	^579^
	Citral	Oropharyngeal dysphagia	Improved swallowing response	Phase II	Gastrointestinal disorders (diarrhea and vomiting)	Nestlé Research	NCT02193438	^579^
	A-967079	Acidosis-induced pain	/	Phase II	No reduction in pain	The University of Erlangen-Nürnberg	The Ethics Committee of the University of Erlangen-Nürnberg (23-16B)	^713^
	Cinnamon, ginger, capsicum	Muscle cramp	A small short-term effect on the cramp threshold frequency	Phase II	There was no effect on cramp intensity, or perceived pain before and after electrically-induced muscle cramps	/	DRKS00009488	^714^
TRPM8	PF-05105679	Cold-related pain	/	Phase I	Oral hypoesthesia, somnolence, hot feeling	Pfizer Limited	NCT01393652	^575^
	L-menthol	Cutaneous pain and hyperalgesia, COPD	Dyspnea relief	Phase II	Did not decrease neural respiratory drive or modulate the breathing pattern/timing in the COPD group	/	UMIN Clinical Trials Registry; No. UMIN000033822 NCT04711044	^715^
	Menthol	Pain, urinary bladder disorders, cough, dry eye disease, type 2 diabetes, obesity	Increase core body temperature and metabolic rate, cooling, analgesic	/	Short-lived effect, dependency, addiction	/	NCT03610386	^716,717^
	Peppermint oil	Neuropathic pain	Pain relief	/	The treated skin had started reddening	University College London Hospitals	/	^718^
	AMG 333	Migraine	/	Phase I	Increased blood pressure	Amgen	NCT01953341	^719^
	DIPA	Sigmoid cancer	Regulation of colonic motility	/				^720^
	Cryosim-1	Itch	/	Phase II	/	/	/	^570^
	Isopulegol	Oropharyngeal dysphagia	Improved swallowing response	Phase II	Gastrointestinal disorders (diarrhea and vomiting)	Nestlé Research	NCT02193438	^579^
	Cool temperature	Colonic peristalsis	Inhibits colonic peristalsis	Phase I	None	/	No. UMIN-CTR; UMIN000030725	^721^
	Cryosim-3	Dry eye discomfort (DED)	Relieves DED symptoms, aids tear production, cooling sensation	Phase I	Temporary eye discomfort	/	ISRCTN24802609 ISRCTN13359367	^722^
TRPM2	/	Bipolar disorder (BD)	Genetic variants in the *TRPM2* gene increase the risk of BD	/	/	University of Toronto	The Research Ethics Board of the Centre for Addiction and Mental Health	^723^
TRPM3	/	Developmental and epileptic encephalopathies (DEE), intellectual disability, epilepsy	De novo mutations in *TRPM3* cause mental retardation and epilepsy	/	/	GeneDx	SCV000891785 SCV000891786	^724^
	μOR, NTX, PregS, ononetin	Myalgic encephalomyelitis/chronic fatigue syndrome (ME/CFS)	Pain relief, immune regulation	Phase II	/	/	The Griffith University Human Research Ethics Committee (HREC/15/QGC/63)	^725^
TRPC4/5	TRPC4/5 inhibitor	Anxiety, depression	/	Phase I	/	Hydra Biosciences; Boehringer Ingelheim	/	^570^
TRPC6	GsMTx-4	Chemotherapy-related heart failure	Reduced doxorubicin-induced apoptosis and cardiotoxicity	Phase III	/	Mayo Clinic	The institutional review board at the Mayo Clinic	^726^

TRPV1 agonist: resiniferatoxin, capsaicin; TRPV1 antagonists: AZD1386, JNJ-39439335, NEO6860, SB-705498, XEN-D0501, PAC-14028, AMG517, ABT-102, BCTC; SYL1001, a short interfering (si) RNA targeting TRPV1; TRPV4 antagonists: GSK2798745; TRPV3 agonist: eugenol, carvacrol; TRPV3 antagonists: GRC15300

TRPA1 agonist: methylglyoxal, cinnamaldehyde, citral, cinnamon, ginger, capsicum; TRPA1 antagonists: GRC1753689, GDC-0334, ODM-108, A-967079

TRPM8 agonist: L-menthol, menthol, peppermint oil, DIPA, Cryosim-1, isopulegol, Cryosim-3; TRPM8 antagonists: PF-05105679, AMG 333; TRPM3 antagonists: μOR, NTX, PregS, ononetin; TRPC6 antagonists: GsMTx-4

P2X3 antagonists: AF-219. The TRPV4-ATP-P2X3 axis is a driver of airway sensory reflexes such as coughing

The specificity of TRP channel agonists and antagonists needs to be enhanced. As some TRP modulators, such as hydroxy-α-sanshool, activate not only TRPA1, but also TRPV1.^615^ Voacangine is an antagonist of TRPV1 and TRPM8, but is also an agonist of TRPA1.^616^

Efficient and specific modulation of TRP channels is a promising to be a new approach to the treatment of various diseases in the future. The above-mentioned adverse events should be considered in clinical studies.

## Conclusions and perspectives

With rapid advances in the field of TRPs, numerous publications have demonstrated that TRPs function as important channels intracellularly, and their abnormalities serve as a pathological triggers for diseases. Herein, we summarized all types of TRPs and demonstrated their functions in multiple biological processes. Furthermore, this review summarized the structures and therapeutic interventions and limitations of TRPs from a historical perspective. However, a number of adverse events remain to be addressed in this area, such as fever, pain, nausea, and even lack of therapeutic efficacy in clinical trials of some studies. The cause of these adverse events may be due to the wide distribution of TRP channels in the body, and modulation of TRP channels can cause systemic reactions. In addition, the specificity of existing drugs for TRP channels needs to be enhanced. To overcome some of these side effects, low doses, topical treatment and precise targeted drug delivery can be used.

To address the issue of precise targeted drug delivery, scholars must ensure the specificity of developed drugs for TRP channels, which will reduce cross-infection caused by TRP channels and reduce damage to other tissues. Second, there is a need to clarify the mechanisms by which drugs modulate TRP channels must be clarified. Finally, new and more effective compounds that can reduce the amount of drugs used must be developed.

Improving the targeting of existing drugs, nano-delivery systems are an interesting topic. In future research, on the one hand, the modification of nanoparticles with TRP channel modulators can be explored in order to exert targeted effects. On the other hand, given that graphene can be combined with metal nanostructures, it can the ability to absorb light by relying on the friction of carbon atoms to generate heat and thus modulate thermally activated TRP channels. Both directions are yet to be examined.

In clinical practice, a mixture of modalities is commonly used to treat diseases. Therefore, a combination of physical interventions and drugs can be proposed to exert a synergistic or amplifying effect on the treatment of diseases associated with TRPs. Multiple treatment combinations can be used with a combination of Western and Chinese medicine. Chemical drugs and physical stimulation methods, such as acupuncture and electroacupuncture are used together. We predict that new therapeutic modalities such as hyperthermia, cold compresses, drug delivery, targeted, Synergistic treatment with multiple modalities, and gene based therapies will be increasingly explored in the future for the treatment of human TRP channel-related diseases.
